# Telomere‐to‐Telomere Genomes Reveal that Multiscale Evolution Shapes the Largest Metabolic Arsenal of *Diaporthe* Fungi

**DOI:** 10.1002/advs.202513287

**Published:** 2026-03-13

**Authors:** Kainan Li, Chen Zhang, Zhichao Zhang, Jiaheng Cheng, Qiuyan Gao, Long Gao, Xiaolin Zhao, Wei Yan, Yuanchao Wang, Wenwu Ye

**Affiliations:** ^1^ State Key Laboratory of Agricultural and Forestry Biosecurity College of Plant Protection Nanjing Agricultural University Nanjing Jiangsu China; ^2^ Key Laboratory of Soybean Disease and Pest Control (Ministry of Agriculture and Rural Affairs) Nanjing Agricultural University Nanjing Jiangsu China

**Keywords:** chromosomal rearrangement, comparative genomics, Diaporthe fungi, secondary metabolite biosynthetic gene clusters, virulence evolution

## Abstract

The fungal genus *Diaporthe* poses a significant threat to global food security by causing devastating crop diseases, including soybean seed decay and stem blight caused by *D. longicolla*. However, the molecular basis of its pathogenicity and the evolutionary mechanisms underlying its virulence remain poorly understood. Here, we present complete telomere‐to‐telomere genome assemblies of four *Diaporthe* species, revealing extensive chromosomal rearrangements correlating with phylogenetic divergence. Comparative analyses of 34 *Diaporthe* genomes identified secondary metabolism genes as the most variable fraction. Comprehensive genome exploration across fungi has revealed that *Diaporthe* harbors the largest repertoire of secondary metabolite biosynthetic gene clusters (SMBGCs) reported to date. We demonstrate that frequent chromosomal rearrangements and rapid intra‐cluster gene variation are key drivers of SMBGC diversification, thereby accelerating the evolution of these gene clusters. Interestingly, we identified horizontal gene transfer events that further expanded the metabolic potential of these clusters. Functional characterization of the five rapidly evolving SMBGCs identified demonstrated their direct role in mediating pathogenicity, underscoring the biological significance of their rapid diversification. Collectively, this study establishes chromosomal plasticity as a crucial mechanism for ecological adaptation and secondary metabolite arsenal expansion in plant pathogens, providing new insights into the evolution of fungal virulence.

## Graphical abstract

This study presents the first telomere‐to‐telomere genomes and population resources for *Diaporthe* pathogens, uncovering the largest known fungal repertoire of secondary metabolite gene clusters. Structural variations and horizontal gene transfer drive cluster diversification, while specific rapidly evolving clusters control virulence, offering novel mechanistic insights into fungal adaptive evolution and pathogenicity.

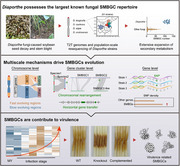

## 1 Introduction

*Diaporthe* (anamorph *Phomopsis*) is a fungal genus that includes plant pathogens, endophytes, and saprophytes [1, 2]. Some species in this genus can also pose threats to mammalian health, including human health [1]. *Diaporthe* pathogens cause a wide range of plant diseases (e.g., cankers, diebacks, rots, blights, and wilts), particularly in economically important crops, such as grapevines, sunflowers, citrus, and soybeans [3, 4, 5, 6]. Notably, *D. longicolla*, *D. unshiuensis*, *D. sojae*, and *D. caulivora* are major soybean pathogens that cause seed decay, pod and stem blight, and stem canker, and leading to significant yield losses worldwide [6]. For example, it was estimated that *Diaporthe*/*Phomopsis* complex‐caused seed decay caused more than 87 million bushels of yield loss in 2018 in the United States and Ontario, Canada [7]. Despite the agricultural impact of *Diaporthe*, the pathogenic mechanisms of these fungi remain poorly understood. Moreover, despite the fact that recent studies revealed that *Diaporthe* produces a diverse array of secondary metabolites (SMs) that appear to be closely associated with fungal pathogenesis, the biosynthetic potential of *Diaporthe* and the underlying mechanisms regulating SM production remain largely unknown [8, 9].

SMs play diverse roles in fungi, functioning as toxins, antibiotics, antifungal agents, and enzyme inhibitors [10, 11]. In many plant‐pathogenic fungi, SMs act as critical signals or toxins that mediate host‐pathogen interactions [12, 13]. Approximately 33500 bioactive microbial metabolites have been described, 47% of which are produced by fungi [14]. Notably, many of these fungal metabolites are unique to the genus *Diaporthe* [15, 16]. The SMs reported from *Diaporthe* exhibit a wide range of bioactivities. For example, diaporthelactone, 7‐methoxy‐4,6‐dimethyl‐3H‐isobenzofuran‐1‐one, and mycoepoxydiene, identified from *Diaporthe* sp., show cytotoxic activity, whereas five cadinene sesquiterpene derivatives isolated from *D. cassiae* possess antifungal activity [17, 18]. In addition, four plant toxins—convolvulanic acid A, convolvulanic acid B, convolvulol, and the α‐pyrone convolvulopyrone — were recently isolated from *D. convolvulus* [19]. Despite the discovery of numerous *Diaporthe* SMs, a systematic genome‐wide analysis of the biosynthetic potential and pathways involving these SMs in this genus has not yet been reported.

SMs are ultimately derived from central metabolic pathways and primary metabolite pools [20]. Interestingly, the biosynthetic genes responsible for SM production are typically organized into clusters within fungal genomes [11, 21]. These secondary metabolite biosynthetic gene clusters (SMBGCs) usually contain core genes encoding enzymes that assemble the chemical backbone of each SM (e.g., nonribosomal peptide synthetases, polyketide synthases, or terpene cyclases) [22, 23]. In addition to these core biosynthetic enzymes, SMBGCs generally include genes encoding oxidases, transporters, regulatory proteins, and transcription factors [24]. The clustered organization of SMBGCs is thought to facilitate the evolution of novel metabolites via multiple mechanisms. At the gene level, the specific composition and polymorphisms of biosynthetic genes can lead to structural variations in the resulting SMs [25, 26, 27]. At the cluster level, gene duplication, deletion, or pseudogenization events can give rise to new metabolic capabilities [28, 29]. Consistent with this, fungal populations often exhibit substantial genetic polymorphisms in SMBGCs, which directly contributes to SM diversity [30]. The evolutionary origins of SMBGCs themselves remain a subject of debate, with hypotheses that include horizontal gene transfer (HGT) and other genetic relocalization mechanisms [27]. However, while loss‐of‐function mutations have been well documented in fungal populations (owing to their discernible phenotypes), the origins and diversification of SMBGCs remain poorly understood [30]. Moreover, most prior research on SMBGC evolution has focused on individual clusters, with few studies examining genome‐wide variations in SMBGC content within species [31].

Fungal genomes typically encode dozens of SMBGCs, and each cluster can range from as few as 2 genes to over 20 genes [20]. The composition of SMBGCs varies greatly across species, likely reflecting their adaptation to different ecological niches [10]. Even among closely related species, the total number of SMBGCs can vary substantially [32]. The remarkable richness of SMs in *Diaporthe* species, coupled with the partially overlapping metabolite profiles among such species, makes this genus a particularly compelling model for studying SM diversity and evolution. Historically, SM discovery involved the extraction of compounds from natural sources, followed by chemical isolation, purification, and bioactivity testing. This traditional method has increasingly been supplemented by genome sequencing and computational mining of genomic and metagenomic data to predict and identify SMBGCs [33]. Consequently, the quality and completeness of the genome sequences are critical for SMBGC research. Although genome sequences for numerous *Diaporthe* species have been publicly released, existing genome assemblies remain fragmented and incomplete, particularly in repetitive and subtelomeric regions, significantly restricting precise evolutionary analyses of SMBGCs. Recent studies of other plant pathogens have demonstrated the effectiveness of telomere‐to‐telomere (T2T) genome assemblies in overcoming these challenges. Notably, T2T assemblies have revealed that chromosome fusion events drive effector gene emergence and adaptation in the subtelomeric areas, thereby offering insights into the structural foundation of virulence evolution in oomycetes [34]. Such discoveries underscore the pivotal role of T2T assemblies in elucidating the genomic dynamics of pathogenicity. However, no complete T2T genome assembly is currently available for *Diaporthe*, which impedes comprehensive genomic research in this genus.

In this study, we generated T2T genome assemblies for four *Diaporthe* species, *D. longicolla*, *D. unshiuensis*, *D. sojae*, and *D. caulivora*, using PacBio HiFi and Oxford Nanopore Technologies (ONT) long‐read sequencing combined with optimized assembly strategies. Our genomic analyses revealed that *Diaporthe* harbors the most extensive repertoire of SMBGCs recorded to date in fungi. Interestingly, these SMBGCs are preferentially located in rapidly evolving genomic regions and subjected to positive selection. Comparative genomics further showed that frequent chromosomal rearrangements in *Diaporthe* drive the diversification and rapid evolution of SMBGCs. Furthermore, we identified five virulence‐associated gene clusters that may synthesize novel compounds to facilitate plant infection.

## 2 Results

### 2.1 T2T Genome Assemblies Reveal Complete Chromosomal Landscapes of *Diaporthe*

**FIGURE 1:**
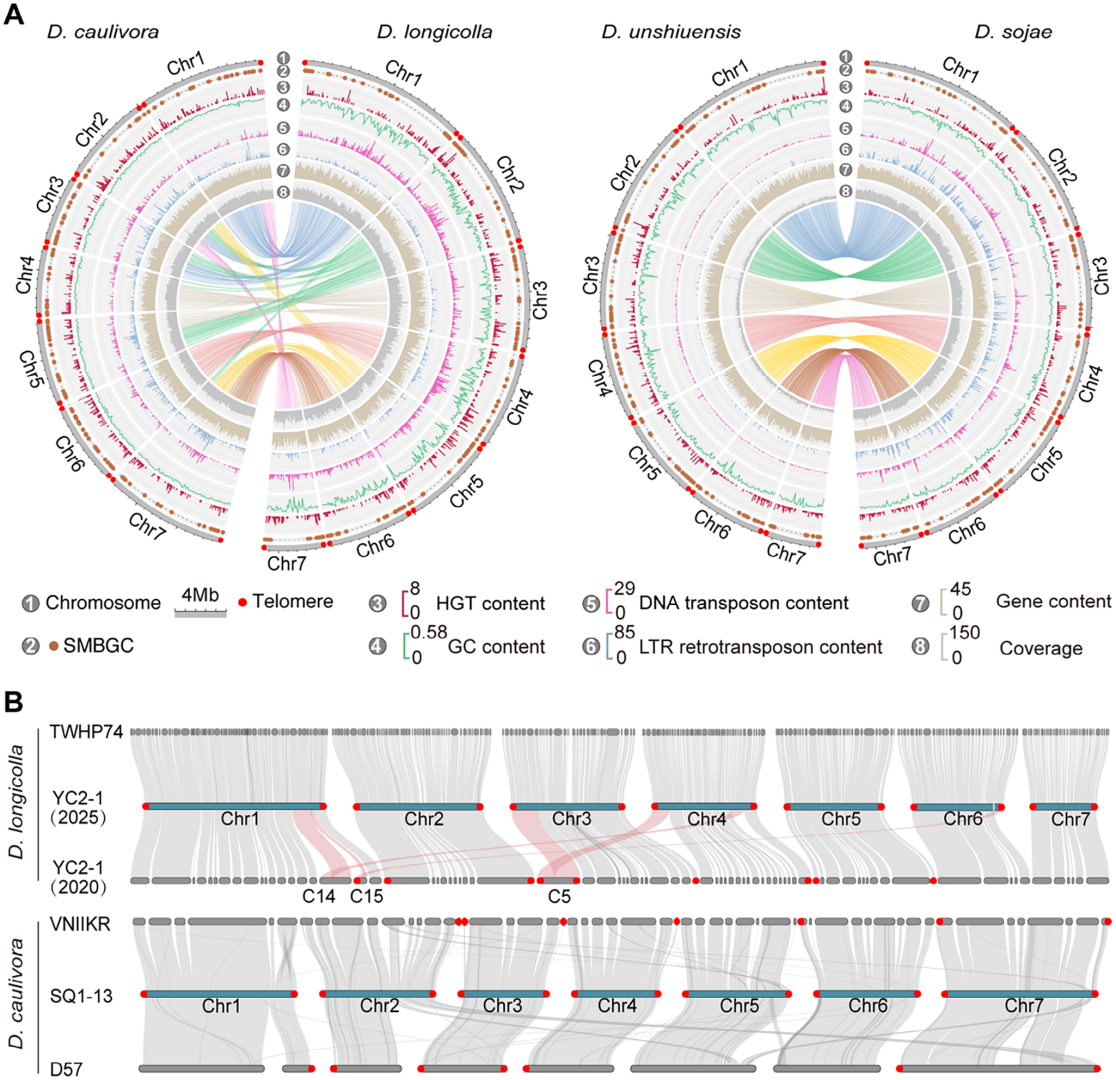
Characteristics of four *Diaporthe* T2T genome assemblies. (A) Circular schemes were used to illustrate the genomic features of four *Diaporthe* T2T assemblies. Circular layers represent the following (from outside to inside): 1. Chromosomes (minimum scale 50 kb); 2. Secondary metabolite biosynthetic gene clusters; 3. Gene content from horizontal transfer; 4. GC content; 5. DNA transposon content; 6. LTR retrotransposon content; 7. Gene content; 8. Average coverage per base in each window. Data were analyzed using 50‐kb genomic windows. (B) Collinearity and telomere distribution comparisons among *D. longicolla* YC2‐1 (2025), *D. longicolla* YC2‐1 (2020), and *D. longicolla* TWP74 (upper panel) and *D. caulivora* SQ1‐13, *D. caulivora* D57, and *D. caulivora* VNIIKR (lower panel). *D. longicolla* YC2‐1 (2025) and *D. caulivora* SQ1‐13 genomes were assembled in this study.

To obtain high‐quality reference genome assemblies of *Diaporthe*, four representative species strains, namely *D. longicolla* (YC2‐1), *D. unshiuensis* (XZ3‐S‐5), *D. sojae* (DT‐S2‐18), and *D. caulivora* (SQ1‐13), were sequenced (Figure 1A). A hybrid sequencing strategy combining PacBio Sequel II HiFi and ONT ultra‐long reads was used to assemble the genome of *D. longicolla* (YC2‐1), the major pathogen associated with soybean seed decay. The other three species genomes were assembled using PacBio HiFi reads only. Each of the genomes of all four species was successfully assembled into seven complete T2T chromosomes, each of which were characterized by canonical telomeric repeat sequences (5’‐CCCTAA‐3’) at both termini (Figure 1A). To visualize the genomic architecture, we computed the density distributions of GC content, genes, transposable elements (TEs), and sequencing read coverage using 50‐kb sliding windows along each chromosome (Figure 1A).

The newly assembled *D. longicolla* YC2‐1 (2025) genome showed good global collinearity and conserved telomere organization compared to the published *D. longicolla* YC2‐1 (2020) assembly (Figure 1B) [35]. In particular, several assembly errors in the regions C14, C15, and C5 of the previous version, indicated by an abnormal reduction in read coverage, were corrected, and these regions significantly improved read depth in the new assembly (Figure 1B; Figure S1). Similarly, the genome of *D. caulivora* SQ1‐13 showed good synteny and conserved telomere structures when compared with the other genomic resources of this species (Figure 1B). Collectively, these results strongly demonstrated that these new T2T‐level assemblies show great improvements in terms of completeness, accuracy, and continuity compared to previous genomic resources, thus providing robust reference genomes for future comparative and functional studies of *Diaporthe*.

### 2.2 Chromosomal Rearrangements Correlate with Genetic Diversity among *Diaporthe* Species

**FIGURE 2:**
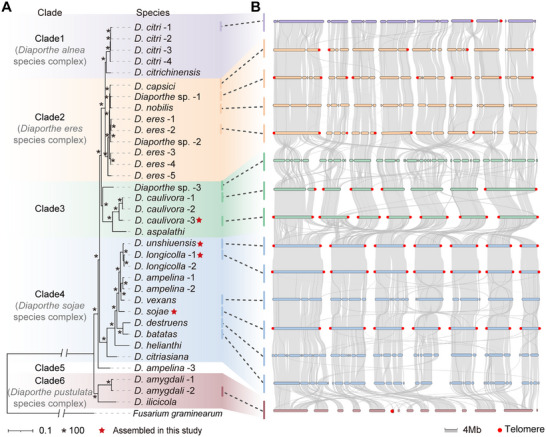
Chromosomal rearrangements and evolutionary relationships among *Diaporthe* species. (A) Phylogenetic tree based on 3174 single‐copy orthologs of 34 *Diaporthe* strains from 21 species; *Fusarium graminearum* was used an outgroup. Bootstrap values for maximum likelihood, equivalent to 100%, are indicated by asterisks on tree branches. (B) Chromosome‐level homology analyses among *Diaporthe* genomes with high‐quality assemblies.

To elucidate phylogenetic relationships and genomic diversity within the genus *Diaporthe*, we conducted a comprehensive genomic analysis of 34 strains spanning 20 distinct species, including 4 genomes newly assembled in this study and 30 publicly available genomes retrieved from the NCBI database (Table S1). Genome assemblies varied considerably in quality, ranging from 7 to 7376 scaffolds or contigs, but consistently exhibited high completeness (>96%) based on BUSCO assessments for 3817 conserved single‐copy orthologous genes (Figure S2). Genome sizes ranged between 47.33 and 65.22 Mb, while gene counts fluctuated between 12903 and 18154 (Figure S2). Notably, genome size was positively correlated with repetitive sequence content (r = 0.74, *p* < 0.001), whereas no significant correlation was observed with gene number (Figure S3). Previous classifications have divided *Diaporthe* genus members into 13 species complexes, including the *D. sojae* complex (containing *D. unshiuensis* and *D. sojae*), whereas the taxonomic placement of *D. longicolla* and *D. caulivora* remains unresolved [36]. Phylogenetic analyses using 3174 single‐copy orthologous genes demonstrated that *D. longicolla* is closely related to *D. unshiuensis* and *D. sojae*, supporting its inclusion in the *D. sojae* complex (Figure 2A). Conversely, *D. caulivora* and *D. aspalathi* formed a distinct clade (Figure 2A). Our analyses support the organization of the 34 *Diaporthe* strains into 6 clades. Substantial genomic variation was observed both between and within these clades, particularly regarding genome size, gene composition, and repetitive element content (Figure S4). These genomic differences likely reflect adaptive evolutionary processes driven by the broad host range characteristics of *Diaporthe*.

Genomes with relatively high assembly quality, each consisting of fewer than 80 contigs, were selected for detailed synteny analyses. The results suggested that *Diaporthe* species possess seven chromosomes (Figure 2B). Genomic synteny analysis further demonstrated extensive chromosomal rearrangements among different species, with particularly striking differences between clades (Figure 2B). Specifically, genomes within the same clade, like those within clade 4, exhibited highly conserved synteny with only minor local variations, whereas substantial chromosomal rearrangements were detected among genomes from distinct clades, for example, between those from clade 3 and clade 4. Notably, the extent of chromosomal structural variations showed a significant correlation with the phylogenetic distances between species (Figure 2A,B). These findings suggest that chromosomal rearrangements are associated with phylogenetic divergence within the *Diaporthe* genus.

### 2.3 *Diaporthe* Possesses an Exceptionally Diverse Secondary Metabolic Biosynthetic Repertoire

**FIGURE 3:**
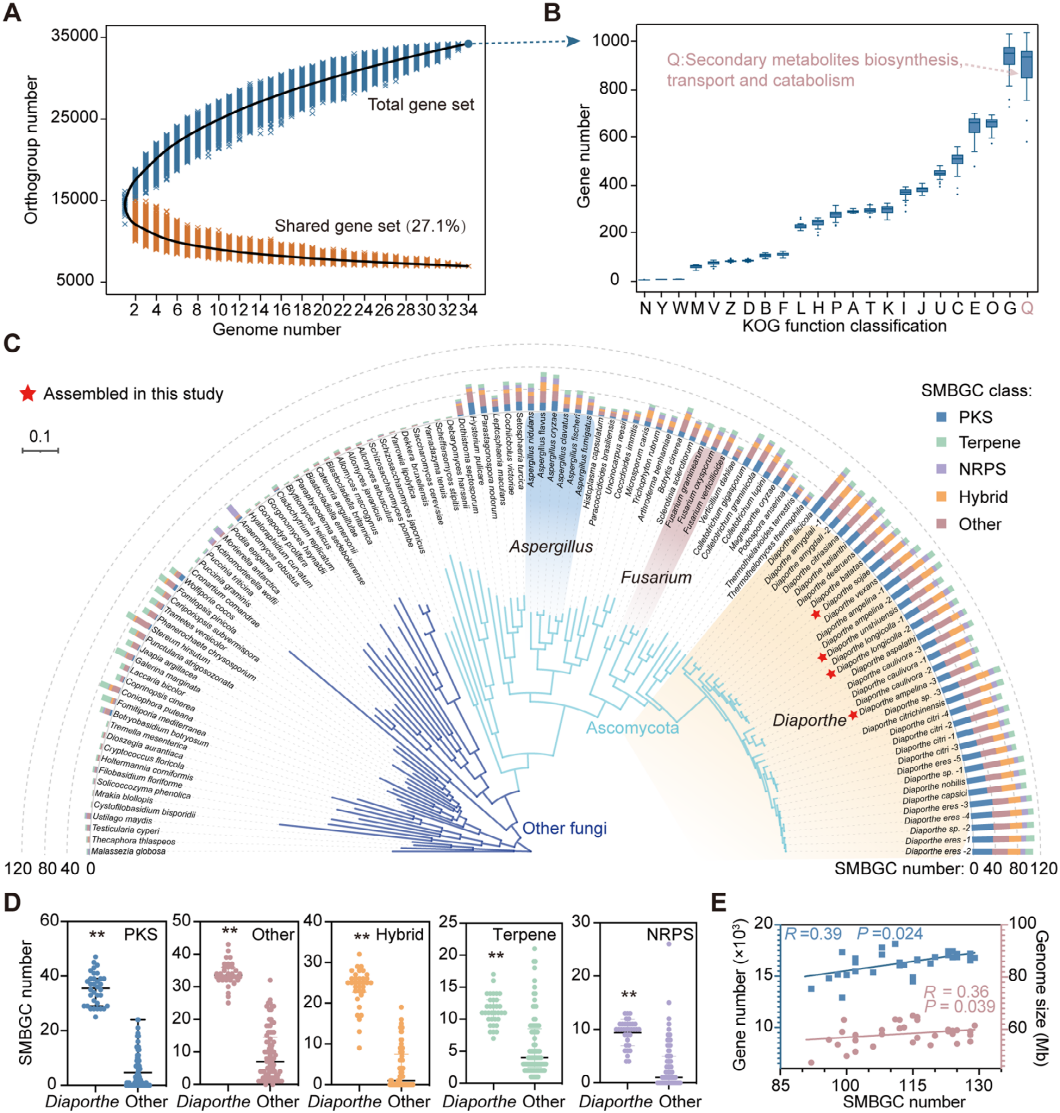
Evidence of an extensive expansion of secondary metabolism genes in *Diaporthe* genomes. (A) Variation of orthogroups in the total gene set and shared gene set along with an additional *Diaporthe* genome. (B) Distribution of proteins allocated to different subsystems as defined by euKaryotic Orthologous Groups (KOGs). KOGs have been sorted according to standard deviations in the gene repertoire. (C) Predicted secondary metabolite biosynthetic gene clusters (SMBGCs) across representative fungal genomes. Phylogenetic tree construction was based on protein sequences from 119 fungal species using orthofinder. “Hybrid,” SMBGCs containing core genes from multiple secondary metabolite families. (D) SMBGC abundance in *Diaporthe* (*n* = 34) compared to that in other fungi (*n* = 85). Data were presented as median with interquartile range. Asterisks indicate significant differences (^**^ *p* < 0.01, two‐tailed Mann–Whitney *U* test). E) Correlation between the number of SMBGCs and both genome size and the number of encoded proteins in *Diaporthe* (*n* = 34). *R*, Pearson correlation coefficient.

To characterize the genomic diversity and complete gene repertoire within *Diaporthe*, we performed comparative genomic analyses through protein sequence clustering across 34 genomes. A total of 553656 genes were grouped into 7015 conserved (shared across all species) and 27229 divergent orthogroups (Table S2). These analyses indicated that only 27.1% of the genes comprised the set of conserved genes, highlighting significant compositional divergence among *Diaporthe* species (Figure 3A). Functional annotation using euKaryotic Orthologous Groups (KOG) categories revealed that metabolic genes accounted for 50% of the conserved gene set and 67% of the divergent gene set (Figure S5A,B). Genes associated with secondary metabolism (category Q), which exhibited the highest variability, were predominantly found in the divergent gene set (Figure 3B; Figure S5A,C). A comparative analysis identified 239 gene families that were positively correlated with total gene content (Figure S6A). Moreover, functional enrichment revealed a significant expansion of secondary metabolism‐related gene families, which potentially explains the observed evolutionary plasticity observed within these KOG functional categories (Figure S6B).

To further investigate SM diversity in *Diaporthe*, we systematically identified SMBGCs across 34 *Diaporthe* genomes and 86 phylogenetically diverse fungal genomes (Tables S3 and S4). Comparative genomics analyses revealed that Ascomycota fungi often harbored significantly more SMBGCs than other phyla, with *Diaporthe* exhibiting an exceptional abundance, surpassing well‐known SM producers, such as *Aspergillus* and *Fusarium* (Figure 3C). *Diaporthe* genomes were particularly enriched in polyketide synthase (PKS), terpene synthase (terpene), and nonribosomal peptide synthetase (NRPS) clusters, along with a notable abundance of hybrid SMBGCs relative to other fungal references (Figure 3D). SMBGC abundance correlated positively with total gene count (*R* = 0.39, *p* = 0.024), reinforcing the earlier observation that secondary metabolism‐related gene family expansion is a major contributor to gene content variation (Figure 3E). Collectively, these results highlight *Diaporthe* as a biosynthetic powerhouse among fungi, with expanded secondary metabolic capabilities, and suggesting an untapped chemical diversity worthy of further exploration.

### 2.4 Diversity of SMBGCs Drives Virulence Evolution in *D. longicolla*

**FIGURE 4:**
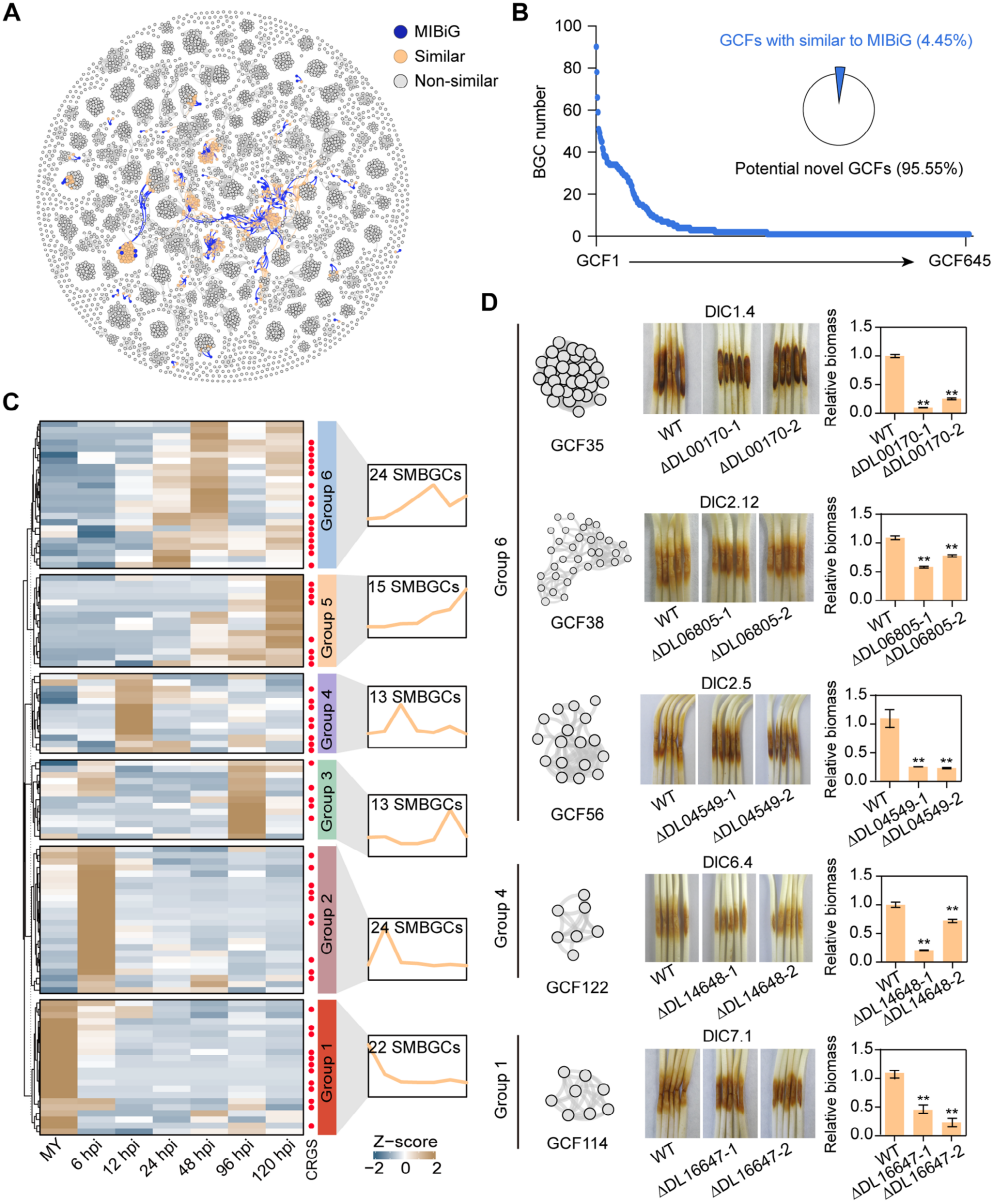
Diverse secondary metabolites act as the virulence factors to promote *Diaporthe* infection in soybean. (A) Similarity network of *Diaporthe* SMBGCs. The network nodes represent individual SMBGCs, categorized as follows: MIBiG (blue), characterized SMBGCs from the minimum information about a biosynthetic gene cluster database; Similar (orange), *Diaporthe* SMBGCs showing homology to characterized clusters; and Non‐similar (gray), *Diaporthe* SMBGCs without detectable homology to characterized clusters (representing potential novel SMBGCs). (B) Distribution and classification of SMBGCs into gene cluster families (GCFs). (C) Transcriptomic analysis of SMBGC expression in *D. longicolla*. CRGS, coordinately regulated gene sets; hpi, hours post‐infection; MY, mycelial stage. The heatmaps show Z‐score standardized TPM values. Expression clusters were defined by the mfuzz algorithm. (D) Five SMBGCs are required for the full virulence of *D. longicolla* YC2‐1 in soybean. Hypocotyls of soybean cultivar Williams 82 were grown at 25°C in the dark and 80% humidity for 4 d before inoculation with mycelia previously cultured in liquid complete medium for 30 h. Pictures were taken, and statistical analyses to determine *D. longicolla* biomass were performed 3 days after inoculation. The pictures show representative disease phenotypes, in which hypocotyls inoculated with SMBGC core gene deletion mutants exhibit reduced lesion length compared with the wild‐type strain (WT). The bar plots represent the relative fungal biomass of *D. longicolla* colonizing soybean hypocotyls. Asterisks indicate statistically significant differences relative to WT is indicated (^**^ *p <* 0.01, two‐sided Wilcoxon tests, *n* = 3).

To explore the biosynthetic diversity and functional potential of SMBGCs in *Diaporthe*, we generated a comprehensive similarity network by integrating clusters from *Diaporthe* with experimentally validated clusters from the Minimum Information about a Biosynthetic Gene cluster (MIBiG) database. Clustering based on conserved biosynthetic architectures identified 645 gene cluster families (GCFs) (Figure 4A; Table S5). Remarkably, half of these clusters were classified as singleton GCFs, and only 4.45% showed similarity to previously characterized SMBGCs, highlighting the unexplored chemical potential of *Diaporthe* (Figure 4B). A comparative analysis of core biosynthetic genes and domains (such as ketosynthase [KS] and condensation [C] domains) demonstrated pronounced sequence divergence, corroborating the exceptional genetic diversity of *Diaporthe* SMBGCs (Figure S7). Notably, applying the same similarity network analysis to other fungi revealed that comparable SMBGC diversity is widespread across fungi, indicating that such diversity represents a common feature of fungal secondary metabolism rather than a lineage‐specific trait (Figure S8). Functional annotations identified several pathogenesis‐associated GCFs, including GCF16, which encodes scytalone synthase, an essential enzyme in melanin biosynthesis (Figure S9A). This cluster exhibited notable syntenic conservation across *Diaporthe* species and structural homology with the previously characterized scytalone biosynthetic cluster (BGC0002161) from *Pestalotiopsis fici* (Figure S9B). An evolutionary analysis confirmed the conservation of melanogenic pathway genes in *Diaporthe*, underscoring their biological importance (Figure S9C).

To determine the functionality of SMBGCs during infection, we conducted transcriptomic profiling of the mycelial stage and soybean hypocotyls inoculated with *D. longicolla* at various infection stages. Principal component analysis (PCA) confirmed the high reproducibility of replicate samples (Figure S10A). Weighted correlation network analysis (WGCNA) was employed to identify the patterns of gene expression within SMBGCs (Figure S10B,C). SMBGCs exhibiting coordinated gene expression in >40% of their constituent genes were defined as “coordinately regulated gene sets” (CRGS). WGCNA identified 60 out of 111 SMBGCs as CRGS, distributed across six distinct expression patterns, which were stage‐specific, suggesting that SMBGCs contribute to distinct infection phases and are potentially critical for *Diaporthe* pathogenicity (Figure 4C; Table S6).

To elucidate the roles of SMBGCs in *Diaporthe* virulence, we functionally validated eight highly expressed CRGS gene clusters during infection. These clusters belong to structurally novel GCFs (Figure 4D; Table S5). Further, the core biosynthetic genes within these SMBGCs were disrupted via knockout mutagenesis (Figure S11). Notably, DlC7.1 knockout significantly impaired mycelial growth, whereas the disruption of the other seven SMBGCs did not affect vegetative growth (Figure S12). In contrast, the knockout of five SMBGCs significantly reduced *D. longicolla* pathogenicity to soybean (Figure 4D; Figure S13). Importantly, the pathogenicity defects were fully rescued in the corresponding complemented strains, confirming the causal link between these genes and virulence (Figure S14). Specifically, DlC6.4 (Group 4) exhibited increased expression at 12 h postinoculation, whereas DlC1.4, DlC2.12, and DlC2.5 (Group 6) showed maximum expression levels between 24 and 48 h, suggesting their involvement in synthesizing key metabolites during the early infection stages (Figure 4D). DlC7.1 showed elevated expression during the mycelial stage, which is consistent with its growth phenotype (also restored in the complemented strain), indicating its critical role in *D. longicolla* development (Figure 4D; Figures S12 and S14). Collectively, these findings emphasize the essential roles of diverse SMs in the virulence and pathogenic adaptation of *Diaporthe*.

### 2.5 Positive Selection Drives Rapid Evolution of Virulence‐Associated SMBGCs

**FIGURE 5:**
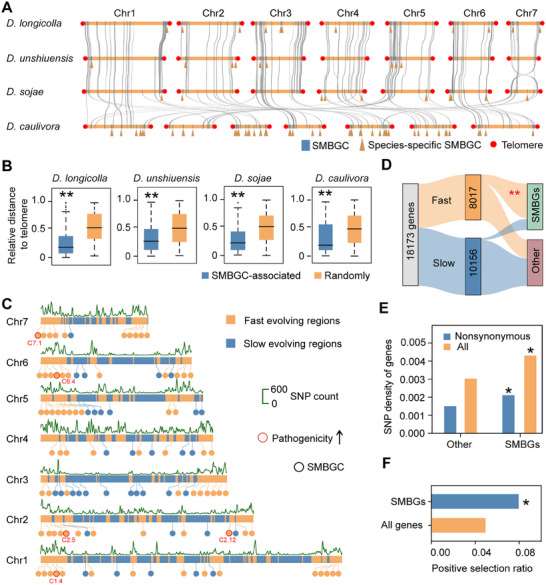
Rapid evolutionary dynamics of virulence‐associated SMBGCs in *Diaporthe*. (A) Chromosomal localization of SMBGCs. (B) Distribution of SMBGCs relative to telomeres. Box plots represent the median and 25th and 75th percentiles, whereas whiskers extend to 1.5 times the interquartile range. Statistical significance is indicated by asterisks (^**^ *p <* 0.01, two‐sided Wilcoxon tests, *n* = 100). (C) *D. longicolla* two‐speed genome partitioning and distribution of SMBGCs. In Red, the pathogenic gene clusters identified in this study. (D) Sankey diagram showing the classification of 18 173 genes into fast‐ and slow‐evolving categories, and further annotation into SMBGCs (green) and other genes (purple). (E) SNP density in genes belonging to SMBGCs and other categories. Orange bars, all SNPs; blue bars, nonsynonymous SNPs. SMBGCs show significantly higher SNP density, particularly for nonsynonymous variants. Statistical significance is indicated by an asterisk (^*^ *p <* 0.05, hypergeometric tests, n = 18173). (F) Positive selection ratio of SMBGCs versus that of all genes. SMBGCs are significantly enriched for positively selected genes. Statistical significance is indicated by an asterisk (^*^ *p <* 0.05, hypergeometric tests, n = 18173).

To elucidate the evolutionary pressures shaping SMBGC diversification, we mapped the genomic coordinates across four representative *Diaporthe* genomes (Figure 5A). Comparative analysis of SMBGC localization patterns revealed significant enrichment in the subtelomeric regions (Figure 5B). Subtelomeric regions are genomic hotspots that exhibit accelerated evolutionary rates owing to repetitive sequence plasticity. This subtelomeric localization indicates an evolutionary mechanism that favors rapid genetic innovation. To investigate the mechanisms of intraspecific variation, we used *D. longicolla* as a model system and explored its two‐speed genome architecture, which is characterized by distinct evolutionary compartments. Genome sequencing of 51 Chinese *D. longicolla* isolates, using YC2‐1 as a reference, identified 127643 SNPs, and generated a detailed variation map (Figure S15A). Gaussian mixture modeling defined two distinct genomic compartments: rapidly evolving regions (188.36 ± 136.63 variants/50 kb) and conserved regions (26.04 ± 18.25 variants/50 kb) (Figure S15B; Table S7). Evolutionary hotspots were predominantly localized in subtelomeric regions, where SMBGCs were significantly enriched (Figure 5C,D). Notably, five virulence‐associated SMBGCs exclusively occupied the rapidly evolving compartments, highlighting a conserved evolutionary strategy that facilitates pathogenic adaptation (Figure 5C). The rapid intraspecific evolution of SMBGCs was manifested by high mutation frequencies, including both synonymous and nonsynonymous mutations that are significantly higher than those observed in other genomic regions (Figure 5E). Further analysis showed that SMBGCs are under strong positive selection (Figure 5F; Table S8). These findings demonstrate that positive selection drives SMBGC hyperevolution in *D. longicolla*, which likely facilitates adaptive evolution.

### 2.6 Chromosomal Plasticity Enables Adaptive Remodeling of SMBGCs

**FIGURE 6:**
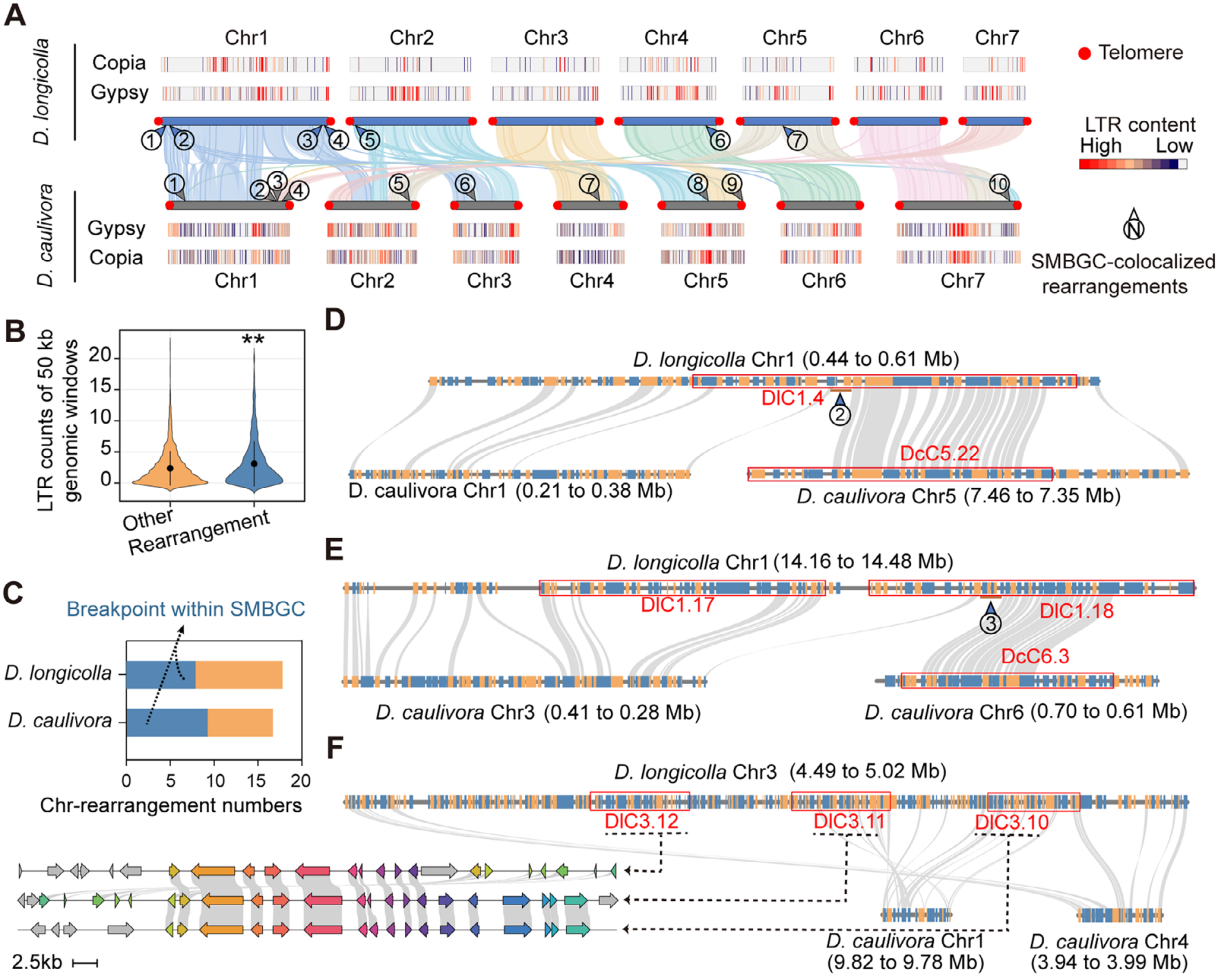
Chromosomal rearrangements drive SMBGCs evolution. (A) Whole genome synteny comparison between *D. longicolla* and *D. caulivora* reveals extensive chromosomal rearrangements. Rearrangements associated with SMBGCs are highlighted and labeled (1–10). Red circles indicate telomeres; *Gypsy* and *Copia* long terminal repeats (LTRs) are shown alongside each chromosome; heatmaps represent local LTR content. (B) Violin plot showing significantly higher LTR retrotransposon density in genomic regions undergoing chromosomal rearrangement compared to other regions (50‐kb windows). Statistical significance (^**^ *p <* 0.01) was determined by two‐sided Wilcoxon tests (n = 2556). (C) Number of chromosomal rearrangement events that intersect with SMBGCs in *D. longicolla* and *D. caulivora*, indicating preferential localization of chromosomal rearrangement at secondary metabolism‐related loci. (D,E) Representative genomic regions illustrating SMBGC‐associated chromosomal rearrangements between the *D. longicolla* and *D. caulivora*. (F) A zoomed‐in view of a lineage‐specific SMBGC insertion and expansion located near the inferred rearrangement breakpoints. Predicted genes on the two strands are shown in orange and blue, respectively; Red boxes indicate SMBGCs disrupted by rearrangements; gray lines denote syntenic relationships.

Species‐specific SMBGC diversity was significantly greater among different species complexes than within individual species complexes, suggesting additional evolutionary mechanisms beyond elevated mutation rates (Figure 5A). Chromosomal rearrangements are structural variations at the chromosomal level that occur when chromosomes break, and the broken ends are abnormally rejoined, often to regions on different chromosomes [37]. The locations at which these breaks occur are referred to as chromosomal breakpoints. Building on the premise that chromosomal rearrangements substantially contribute to *Diaporthe* genetic diversity, we investigated how macroscale genome evolution shapes the architectural plasticity of SMBGCs. Using *D. longicolla* and *D. caulivora* as comparative models, we mapped the structural variations in the genomic regions associated with chromosomal breakpoints (Figure 6A; Table S9). The chromosomal breakpoints exhibited increased densities of TEs, particularly *Copia*‐like and *Gypsy*‐like retrotransposons, indicating retrotransposon‐mediated rearrangements during genome evolution (Figure 6A,B). Noteworthy, SMBGCs significantly colocalized with the chromosomal breakpoints identified in approximately half of the interspecies rearrangement regions (Figure 6A,C).

For instance, the second chromosomal breakpoint identified in *D. longicolla* is located on chromosome 1 and includes the virulence‐associated SMBGC DlC1.4, which corresponds to DcC5.22 on chromosomes 1 and 5 in *D. caulivora* (Figure 6D). Comparative analysis identified partial gene collinearity and revealed a conserved MYB‐type transcription factor harboring three tandem MYB domains within these SMBGCs (Figures S16A,B and S17). Expression analyses indicated a significant upregulation of DlMYB1 during soybean infection by *D. longicolla*, whereas DcMYB1 expression was undetectable under identical conditions (RT‐PCR CT values >35; Figure S16C). This loss of function likely contributes to the attenuated virulence observed in *D. caulivora* (Figure S15D).

Furthermore, our analyses indicated that chromosomal rearrangements is associated with species‐specific SMBGC innovation in *Diaporthe*. For example, breakpoint 3 in *D. longicolla* harbors a species‐specific SMBGC (DlC1.17) (Figure 6E). Notably, Dl3.10, DlC3.11, and DlC3.12 constitute a unique tandem SMBGC cluster that is absent in other *Diaporthe* species and are located in genomic regions proximal to inferred chromosomal breakpoints (Figure 6F). These findings suggest chromosomal rearrangements contribute to species‐specific SMBGC diversification in *Diaporthe*, generating novel metabolic potential through genomic structural innovation.

### 2.7 Horizontal Gene Transfer Enhances SMBGC Innovation in *Diaporthe*

**FIGURE 7:**
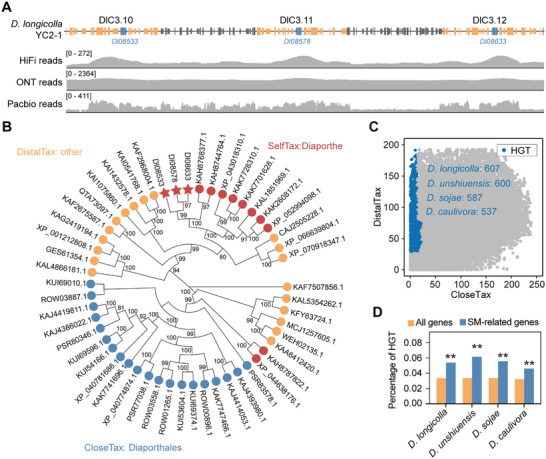
Horizontal gene transfer (HGT) contributes to SMBGC evolution in *Diaporthe*. (a) Coverage plots from three sequencing platforms (HiFi, ONT, and PacBio) support the presence of tandem SMBGCs (DlC3.10, DlC3.11, and DlC3.12) in the genome of *D. longicolla* YC2‐1. The names of those genes putatively acquired by HGT are highlighted in blue. (b) A maximum likelihood phylogeny of a representative gene putatively acquired by HGT was constructed using homologs retrieved from the NCBI nr database. Branch tips are colored by taxonomic assignment: red (self: *Diaporthe*), blue (close taxon: Diaporthales), and orange (distal taxon: other lineages). (c) Genome‐wide identification of genes putatively acquired by HGT across four *Diaporthe* species. Each dot represents a gene, plotted by silhouette score against close (*x*‐axis) and distal (*y*‐axis) taxonomic groups. Genes putatively acquired by HGT candidates (blue) exhibit lower similarity among close taxa and high similarity among distant taxa. (d) Proportion of HGT‐derived genes in the whole genomes versus SMBGCs. SMBGCs are significantly enriched for horizontally acquired genes across all four species. Statistical significance (^**^ *p* < 0.01) was assessed using hypergeometric tests. The sample size corresponds to the total number of genes in the genome‐wide background set.

The genomic region containing the three tandem homologous SMBGCs in *D. longicolla* strain YC2‐1 was absent in strain TWP74. Notably, similar strain‐specific presence/absence variations were also observed in *D. caulivora*, where distinct genomic segments on chromosome 7 were present in strain D57 but absent in other strains (Figure 1B). To validate the authenticity of these SMBGCs, we performed multi‐platform sequencing verification using HiFi, ONT, and PacBio reads. Consistent read coverage across all sequencing platforms confirmed the physical presence of this genomic region in *D. longicolla* YC2‐1, excluding potential assembly artifacts (Figure 7A). Population genomic analyses demonstrated that this SMBGC‐containing region occurred in only a minority of *D. longicolla* strains, with structural deletions prevalent in most isolates (Figure S18), suggesting dynamic evolutionary patterns within the species.

A phylogenetic reconstruction revealed that the three core genes form monophyletic clades with homologs from phylogenetically distant fungi, indicating their potential acquisition through HGT (Figure 7B). Genome‐wide HGT screening across four representative *Diaporthe* species, using Diaporthales as the reference lineage, identified multiple horizontally acquired genes (Figure 7C; Table S10). Horizontally transferred genes exhibited distinct codon usage biases compared to those of native *Diaporthe* genes (Figure S19A). Strikingly, secondary metabolism‐associated genes showed significantly higher HGT frequencies than other functional gene categories (Figure 7D). Both the core biosynthetic genes and tailoring enzymes within the SMBGCs showed HGT signatures (Figure S19B). Phylogenetic donor tracking identified fungi as the predominant contributors to these horizontally acquired secondary metabolism genes (Figure S19C). Furthermore, most SMBGCs contain only a limited fraction of horizontally acquired genes, suggesting that HGT in *Diaporthe* contributes to SMBGC diversification through intra‐cluster innovation rather than whole‐cluster acquisition (Figure S19D). Collectively, our findings established HGT as a key evolutionary mechanism driving SMBGC diversification in *Diaporthe*.

## 3 Discussion

Despite numerous *Diaporthe* genome sequences being publicly available, existing genomic resources remain inadequate to fully capture the extensive phylogenetic diversity of the genus. In this study, we used PacBio HiFi and ONT to generate T2T genome assemblies of *D. longicolla*, *D. unshiuensis*, *D. sojae*, and *D. caulivora*. Additionally, we performed time‐course transcriptomic profiling of *D. longicolla*—the primary pathogen responsible for soybean seed decay—across infection stages and conducted whole‐genome resequencing of 51 geographically diverse strains. These comprehensive datasets significantly improved our comparative genomics, functional genomics, and population genetics studies of the genus *Diaporthe*.

Together, our phylogenetic and comparative genomic analyses of 34 strains from 20 *Diaporthe* species revealed that chromosomal rearrangements are associated with species diversification and represent an important source of genetic variation across distinct species complexes. This variation predominantly results from the expansions of SM pathways. Notably, secondary metabolism emerged as the most genetically diverse cellular subsystem in *Diaporthe*, with genes in SM biosynthesis exhibiting the highest expansion rates. Similar enrichment has been documented in other fungal and plant genomes, suggesting that the acquisition of additional biosynthetic genes represents a rapid and cost‐effective adaptation mechanism [38, 39]. Remarkably, *Diaporthe* possesses a significantly higher number of SMBGCs than the other representative fungal taxa analyzed in this study, underscoring its exceptional biosynthetic potential. SMs play vital ecological roles, including defense, nutrient acquisition, and symbiosis, which enhance the environmental adaptability of organisms [40]. For example, high intraspecific quantitative and qualitative chemotype diversity in plants can allow rapid adaptation to local biotic factors [41, 42, 43], and the ability to produce specific SMs is related to the ecological adaptation and host‐specific interactions of fungi [44]. We hypothesize that the broad host range of *Diaporthe* and its complex ecological interactions drive its extensive SMBGC diversity.

Detailed similarity analyses at the gene cluster, core gene, and domain levels revealed considerable diversity within *Diaporthe* SMBGCs. Only 4.45% of these gene clusters shared similarities with previously characterized clusters, highlighting the potential of this genus to synthesize novel bioactive compounds. The coordinated expression of genes within clusters is a hallmark of functional gene clusters in fungi [45]. As previously mentioned, the term CRGS describes clusters of genes in SMBGCs that exhibit consistent RNA expression patterns [45]. Transcriptomic profiling during *D. longicolla* infection demonstrated that more than half of the SMBGCs constitute CRGS, implying a coordinated production of diverse SMs during infection. Disruption of core genes in these SMBGCs abolished fungal pathogenicity, underscoring the pivotal role of secondary metabolism in *Diaporthe* virulence.

Moreover, our intraspecific comparative analyses of *D. longicolla* suggested a two‐speed genome evolutionary model, with SM‐related genes enriched in rapidly evolving genomic compartments subjected to positive selection. Chromosomal positioning and genome architecture critically influence SMBGC evolution, with SMBGCs preferentially localizing in the subtelomeric regions. Consistent with findings in other eukaryotes, subtelomeric regions represent highly dynamic genomic hotspots that facilitate the rapid evolution of gene families involved in host immune evasion or ecological adaptation [46, 47]. The origins and evolution of fungal SMBGCs remain areas of active investigation, with current hypotheses implicating genomic repositioning and gene duplication mechanisms [27]. Our analyses demonstrate that SMBGC expansions and duplications in *Diaporthe* strongly correlate with chromosomal rearrangements, establishing genomic rearrangements as essential drivers of genetic diversification and SMBGC reorganization. Regions undergoing chromosomal rearrangements frequently exhibit SMBGC emergence, restructuring, or loss, echoing similar observations in *Tolypocladium inflatum* and suggesting that chromosomal plasticity is a universal mechanism in fungal SMBGC evolution [31].

Gene duplication prominently contributes to SM diversification in fungi, whereas HGT occurs intermittently and exhibits distinct species‐specific patterns [48]. We used hit distribution statistics based on sequence homology searches to predict HGTs in *Diaporthe* genomes. The predicted amount of HGTs is comparable to that reported in previous studies [49]. We found that SMBGC‐associated genes were significantly enriched among the candidate HGT regions, reflecting the fact that clustered metabolite genes in fungi are more prone to HGT than unclustered genes [48]. Similarly, in a study on xylarialean endophytes, HGT events were positively correlated with the number of SMBGCs [50].

In summary, our study presents the T2T genome assemblies of multiple *Diaporthe* species, unveiling extensive chromosomal rearrangements across distinct evolutionary clades. Multiomics integration highlights *Diaporthe*’s unparalleled SMBGC diversity among fungi, emphasizing their crucial role in pathogenicity. Our findings further establish that chromosomal plasticity and HGT are the principal drivers of SMBGC structural innovation and diversification, and provide novel insights into the evolutionary dynamics of plant‐associated fungi. Future comparative genomic investigations across broader taxonomic groups will validate these findings, whereas functional genome mining of SMBGCs promises to uncover structurally novel bioactive compounds with agricultural or pharmaceutical potential.

## 4 Conclusion

Our study provides the first T2T genome assembly of four *Diaporthe* species, revealing extensive chromosomal rearrangements and phylogenetic divergence across the genus. Through integrative comparative genomics, transcriptomics, and functional validation, we demonstrate that *Diaporthe* harbors the most diverse array of SMBGCs reported in fungi, many of which directly contribute to pathogenicity. Our findings establish chromosomal plasticity, intra‐cluster gene variation, and HGT as key evolutionary forces driving SMBGC diversification and structural innovation. These mechanisms underlie the metabolic versatility and ecological adaptability of *Diaporthe*, shedding light on the genetic basis of its broad host range and virulence. This work not only advances our understanding of fungal genome evolution and pathogenicity but also provides a valuable foundation for mining novel bioactive compounds with potential applications in agriculture and biotechnology.

## 5 Experimental Section

### 5.1 Genome Sequencing and De Novo T2T Genome Assembly

DNA extracted from the mycelia of *D. longicolla* YC2‐1, *D. unshiuensis* XZ3‐S‐5, *D. sojae* DT‐S2‐18, and *D. caulivora* SQ1‐13 was used for library construction. Qualified libraries were sequenced on a PacBio‐Sequel II platform, while *D. longicolla* YC2‐1 was additionally sequenced using a PromethION platform. Library construction, PacBio HiFi reads, and ONT ultra‐long reads were generated by the Beijing Genomics Institute (BGI‐Shenzhen, China). PacBio HiFi reads were assembled with Hifiasm v0.18.5 and HiCanu v2.2, using the default parameters [51, 52]. ONT ultra‐long reads were assembled using NextDenovo v2.5.0 with default parameters [53]. Both contigs with low GC content (<25%) and mitochondrial genomic DNA were excluded from the analysis. Complete appended telomere sequences were assembled by aligning the contigs generated by Hifiasm with those from HiCanu and NextDenovo. Genome collinearity analyses were performed between genome assemblies generated by different assemblers using TBtools v2.210 and the SynVisio website (https://synvisio.github.io) [54]. Read alignments revealed a high read depth between fragments that lacked connecting evidence, indicating the presence of a large repeat array. Consequently, the gap was filled with 100 unknown bases (N) to bridge the sequences. Finally, this procedure was applied to a single chromosome in each of three genomes: chromosome 7 in *D. longicolla* and *D. sojae*, and chromosome 3 in *D. unshiuensis*. All chromosomes in *D. caulivora* were assembled without gaps. As a result, each genome was assembled into seven chromosomes, with six chromosomes being fully contiguous and gap‐free in the three genomes requiring gap bridging. Several metrics were evaluated to assess the completeness and contiguity of the assembled genome, several metrics were evaluated. Tidk v0.2.65 was used to search for the telomeric sequences [55]. N50 and coverage depth were calculated using QUAST v5.0.256 [56]. Genome assembly completeness was measured using BUSCO v4.1.2, using the Sordariomycetes_odb10 database as a reference [57].

### 5.2 Genome Annotation

Genes were predicted in the genome using Fungap v.1.1.1, a fungal genome annotation pipeline [58]. First, repeat regions were annotated using the existing reference repeat library included in RepeatMasker v.4.1.2, supplemented with novel repeats detected using RepeatModeler v2.0.1 [59]. Genes in the masked genome were predicted using Marker v3.01.03, Augustus v3.4.0, and Braker v2.1.5, which integrates RNA‐seq and high‐quality homologous protein evidence [60, 61, 62]. RNA‐seq reads from the mycelial stage of *D. longicolla* (MY‐1) were used. For the other three *Diaporthe* species, for which species‐specific RNA‐seq data were unavailable, MY‐1 RNA‐seq dataset was used as transcriptomic evidence. RNA‐seq reads were mapped to the individual genome assemblies using HISAT2 v2.0.4 [63]. High‐quality homologous protein evidence was collected from sequences belonging to Swiss‐Prot sequences using DIAMOND v2.1.6.160 [64]. The final gene annotation was an integration of results from the three software programs. The predicted genes were functionally annotated based on the protein sequences queried against the eggNOG 5 database using eggNOG‐mapper v2.1.9 [65]. Long terminal repeat (LTR) retrotransposons were identified and annotated using LTR_retriever v2.9.063 and LTRharvest (GenomeTools) v1.6.264 [66, 67].

### 5.3 Secondary Metabolite Gene Cluster Detection and Clustering

Genome sequences of fungi that were not generated in this study were downloaded from the NCBI database, prioritizing the use of NCBI reference genomes for all comparative analyses (Table S3). To ensure highly accurate SMBGC prediction, we utilized existing NCBI gene annotations as input for SMBGC prediction when they were available for a reference genome. For any reference genomes lacking pre‐existing gene models, de novo gene prediction was performed using Augustus v3.4.0 [62]. All genomes and annotations were submitted in the FASTA and GFF format to antiSMASH fungal version v7 for the detection of SMBGCs [68]. The output was parsed using custom Python scripts to extract comprehensive information regarding the gene clusters (Table S4). Synteny plots were generated using Clinker v1.0 [69]. The SMBGC network was generated using Gephi v0.10.1 (https://github.com/gephi) after pairwise similarity comparison of all SMBGCs predicted by *Diaporthe* and MIBiG databases using BiG‐SCAPE v2.0 (–cutoffs 0.5) [70, 71].

### 5.4 Identification of Horizontal Gene Transfer Regions

HGTector v2, which follows a hybrid approach between “BLAST‐based” and phylogenetic analyses, was putatively horizontally transferred genes in *Diaporthe* [72]. The software was set up with the following stringency parameters: selfTax = 36922, closeTax = 5114, searchTool = diamond, and e‐value cut‐off = 1e‐5 for the BLAST hits, while default values were used for the remaining parameters. The *Diaporthe* predicted proteins were blasted against a local NCBI nr database. The NCBI Taxonomy database (downloaded on February 20, 2022) was used to classify BLAST matches.

### 5.5 Phylogenetic Tree Construction

An evolutionary reconstruction of gene families across 34 *Diaporthe* species was performed using *Fusarium* *graminearum* as the outgroup. The predicted protein sequences were clustered into orthogroups using OrthoFinder v2.5.4, with default settings [73]. For the species tree, multiple sequence alignments for 3,174 single‐copy orthogroups were inferred using Muscle v5.1, followed by the removal of gapped regions from the alignments using Gblocks v0.91b [74, 75]. The species tree was reconstructed in RAxML v8.2.12, with the options of rapid bootstrap analysis and search for the best‐scoring maximum‐likelihood tree activated [76]. This tree was visualized and rooted using ITOL [77].

### 5.6 Comparative Genomic Analyses

Whole‐genome protein synteny alignments were visualized using TBtools v2.210, JCVI, and the SynVisio website (https://synvisio.github.io) [54, 78]. A circos plot was visualized by shinyCircos‐V2.0 [79]. BEDTools v2.29 was used to calculate sliding window size and coverage [80]. Conserved and divergent genomic fractions were defined based on protein groups generated using OrthoFinder v2.5.4 [73]. The growth of the total and shared gene set was assessed by performing 5000 iterations of random genome combinations using a custom Python script. The amplification rate for each gene family was calculated based on the previously mentioned enzyme expansion rate [38]. Selection pressure analysis was conducted based on SNP variation data among *D. longicolla* strains. For each gene, the nonsynonymous (Ka) and synonymous (Ks) substitution rates were calculated using TBtools v2.210, and the Ka/Ks ratio was used to assess selective pressure, with genes showing Ka/Ks >1 considered to be under positive selection [54].

### 5.7 Genome Resequencing Analysis

Library construction was performed using DNA extracted from 51 *D. longicolla* strains isolated in China. Qualified libraries were sequenced on a DNBSEQ‐G400 platform (BGI‐Shenzhen). The alignment software BWA v0.7.10 was used to align the cleaned data from each sample to the reference genome YC2‐1 [81]. SNP and InDel identifications were performed using GATK v3.4.0, following the optimal variant detection analysis protocol recommended on the official GATK website [82]. A phylogenetic tree was constructed based on the SNP matrix of the sample and reference strains using TreeBeST v1.9.2 [83].

### 5.8 RNA Sequencing Analysis

*D. longicolla* YC2‐1 was cultured in complete medium (CM) medium for 3 days and then inoculated into the hypocotyls of soybean (Williams 82) etiolated seedlings cultured in the dark for 5 d. Infecting mycelia were sampled at 0, 6, 12, 24, 48, 96, and 120 h after inoculation. During sampling, the lesion areas of the plants (3 cm long) were intercepted, three soybean seedlings were combined for each sample, and three samples were collected at each stage. Total RNA was extracted from each sample by using a high‐purity total RNA extraction kit (AF505B). The isolated high‐quality RNA was used to construct sequencing libraries, which were further sequenced using a BGIseq500 platform (BGI‐Shenzhen) with a paired‐end sequencing strategy. Clean reads were mapped to the reference genome using HISAT2 v2.0.4 [63]. Samtools, which organizes the comparison results, performs format conversion, and stringently estimates gene expression [84, 85], was also used in our analyses. Both heatmap construction and PCA analysis were performed using TBtools v2.003 [54]. A WGCNA was performed using the R package [86].

### 5.9 *D. longicolla* and Soybean Growth Conditions

*D. longicolla* strain YC2‐1 and all the transgenic strains used in this study were grown on CM agar plates at 28°C in the dark. The growth rate was measured after 3 d of culture. Mycelia were harvested from liquid CM at 28°C and 180 rpm. Soybean (Williams 82) seeds were grown in vermiculite and maintained at 25°C in the dark and 80% humidity for 4 d to harvest the etiolated hypocotyls.

### 5.10 *D. longicolla* Transformation and Infection

*D. longicolla* transformants were generated using PEG‐mediated protoplast transformation as described previously [87]. Positive transformants were verified by PCR. Agarose gel electrophoresis images were color‐inverted for visualization, and bands corresponding to the expected fragment size are shown. The etiolated hypocotyls of soybean seedlings were inoculated with mycelia of either wild‐type or transformant strains, and maintained at 25°C in the dark. Soybean hypocotyls were photographed 3 d after inoculation and collected for biomass determination.

### 5.11 Relative Biomass and Expression Level Determination

Genomic DNA was extracted using a DNAsecure plant kit (TIANGEN Biotech, Beijing, China) following the manufacturer's instructions. Total RNA was extracted from each sample by using a high‐purity total RNA extraction kit (AF505B). qPCR and RT‐qPCR were performed on an ABI 7500 Fast real‐time PCR system (Applied Biosystems Inc., Foster City, CA, USA) using a ChamQ Universal SYBR qPCR Master Mix (Vazyme Biotech Co., Ltd., China) and the primers listed in Table S11, as needed. Data were analyzed using the 2^−ΔΔCT^ method.

### 5.12 Statistics Analysis

Statistical analyses were conducted using the R software, GraphPad Prism, and Excel. For experimental data, unless otherwise specified, results are presented as mean ± standard error of the mean (SEM). For fungal biomass assays, colony growth measurements, pathogenicity tests, and gene expression analyses, data were obtained from three independent biological replicates (*n* = 3). Comparisons between two groups were performed using two‐sided Wilcoxon rank‐sum tests. No data were excluded. The design and data collection of the experiments were randomized, and the infection assays were conducted blinded. The results of all key experiments were reproducibly confirmed. For comparisons of SMBGC abundance between *Diaporthe* and other fungal taxa, a two‐tailed Mann–Whitney *U* test was applied. Correlations between SMBGC number and genome features were assessed using Pearson correlation analysis. For enrichment and proportion analyses, including SNP enrichment, positive selection enrichment, and HGT enrichment in SMBGCs, two‐sided hypergeometric tests were used. In these analyses, the sample size (n) corresponds to the total number of genes in the relevant genome‐wide background set, with the queried subset defined according to the specific analysis described in each figure legend. Differences were considered statistically significant at *p* <0.05.

## Author Contributions

W.W.Y., Y.C.W., and W.Y. designed the research. K.N.L., C.Z., X.L.Z., and J.H.C. prepared the samples. K.N.L., Z.C.Z., C.Z., and L.G. analyzed the data. C.Z., J.H.C., and Q.Y.G. conducted the experiment. and K.N.L. and W.W.Y. wrote the paper.

## Funding

China Agriculture Research System (CARS‐04), National Natural Science Foundation of China (32172374), Fundamental and Interdisciplinary Disciplines Breakthrough Plan of the Ministry of Education of China (JYB2025XDXM703).

## Conflicts of Interest

The authors declare no conflicts of interest.

## Supporting information




**Supporting File 1**: advs74689‐sup‐0001‐SuppMat.docx.

**Supporting File 2**: advs74689‐sup‐0002‐TableS1.xlsx.

## Data Availability

The data that support the findings of this study are available in the supplementary material of this article. The raw sequencing data (genome, RNA and re‐sequencing) and the assembled genome have been deposited in the NCBI BioProject database under accession codes PRJNA1262549, PRJNA1262622, and PRJNA1262630.
