# Supplementary material for: Telomere‐to‐Telomere Genomes Reveal that Multiscale Evolution Shapes the Largest Metabolic Arsenal of Diaporthe Fungi
Source: Adv Sci (Weinh). 2026 Mar 13;13(27):e13287. doi: 10.1002/advs.202513287 (PMC13170202; doi:10.1002/advs.202513287)
Supplement: Supplementary file 1 — Supporting File 1: advs74689‐sup‐0001‐SuppMat.docx. [file ADVS-13-e13287-s001.docx]

Supporting Information

**Telomere-to-Telomere Genomes Reveal that Multiscale Evolution Shapes the Largest Metabolic Arsenal of *Diaporthe* Fungi**

Kainan Li, Chen Zhang, Zhichao Zhang, Jiaheng Cheng, Qiuyan Gao, Long Gao, Xiaolin Zhao, Wei Yan, Yuanchao Wang, and Wenwu Ye*

**
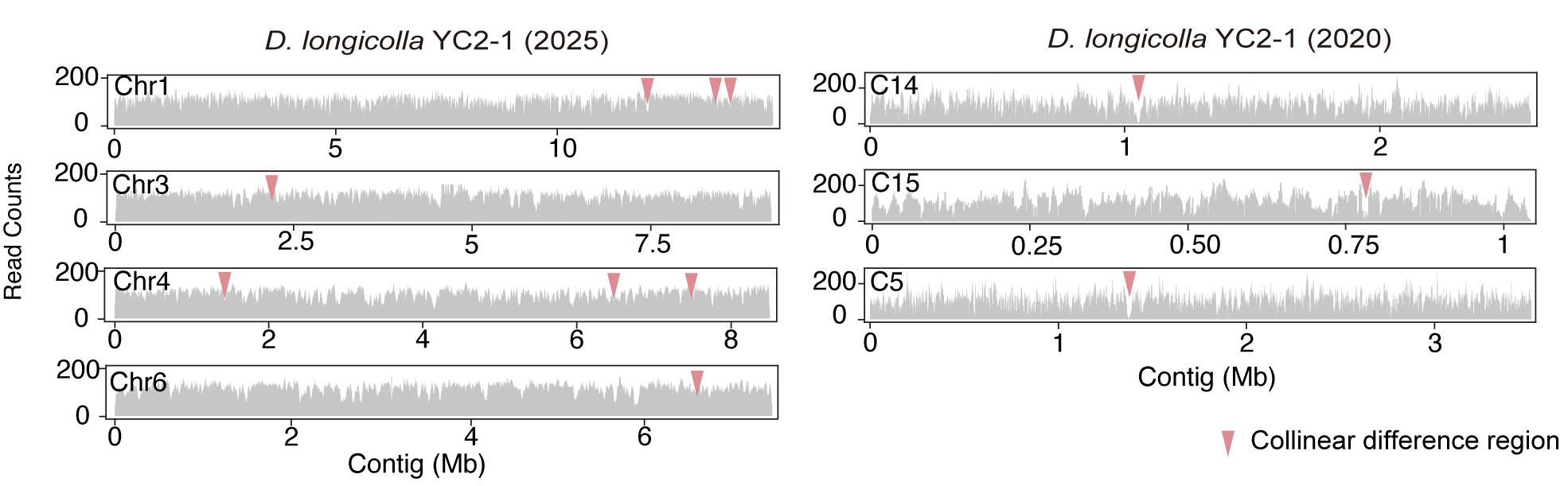
**

**Figure S1. Comparison of read coverage between *D. longicolla* genome assemblies from 2020 and 2025.** Regions with improved assembly quality and read depth are highlighted.


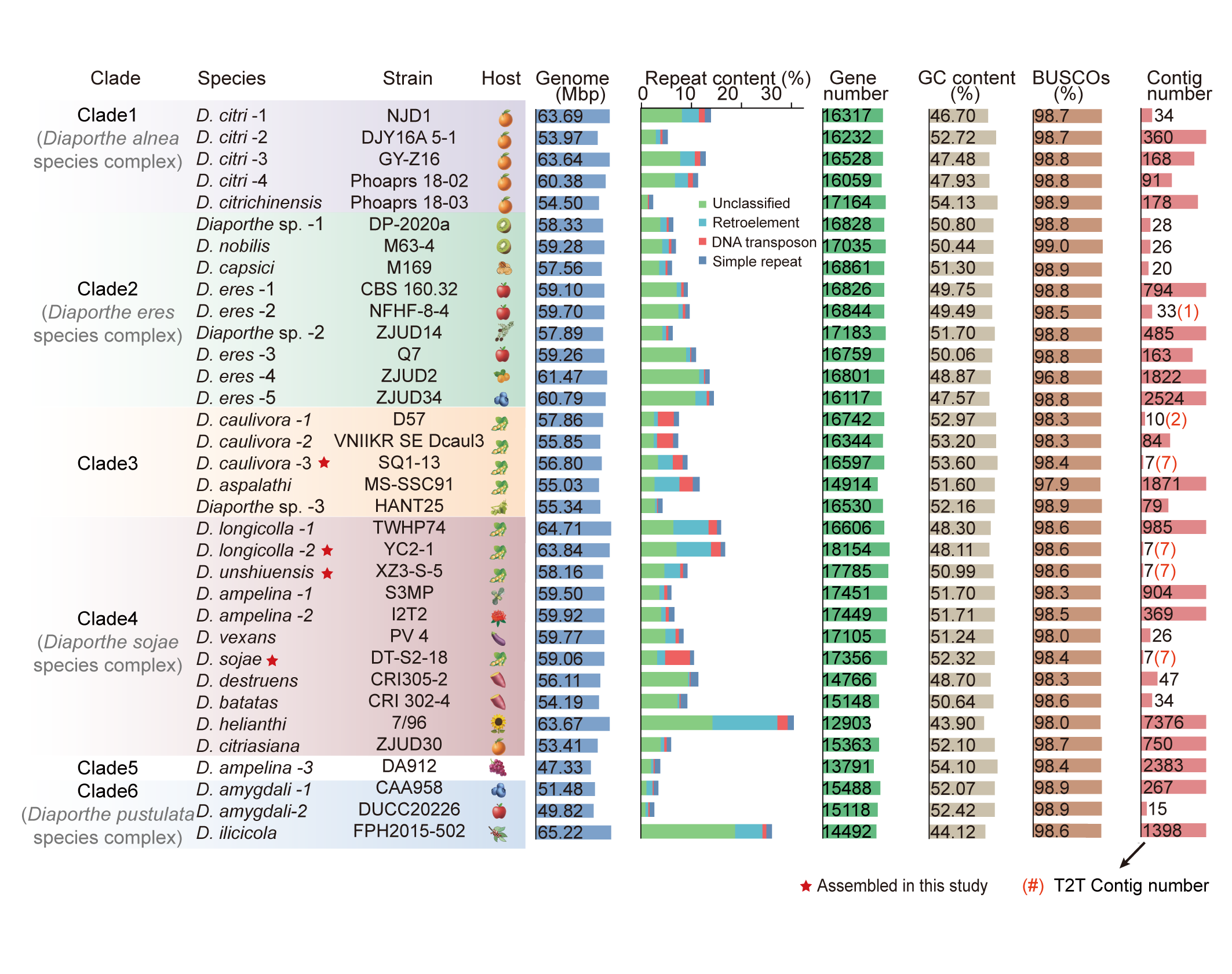
**Figure S2.** **Summary of Genome features and annotation for 34 *Diaporthe* strains.** Strains are grouped by species complex and clade. The table columns detail host association, genome size, repeat content and classification, gene number, GC content, BUSCO completeness, and contig number. Repeat content is categorized into four classes: unclassified, retroelements, DNA transposons, and simple repeats. BUSCO values indicate gene set completeness based on the Sordariomycetes_odb10 database. Red stars denote the genomes assembled in this study; numbers in parentheses represent telomere-to-telomere (T2T) chromosomes.

**
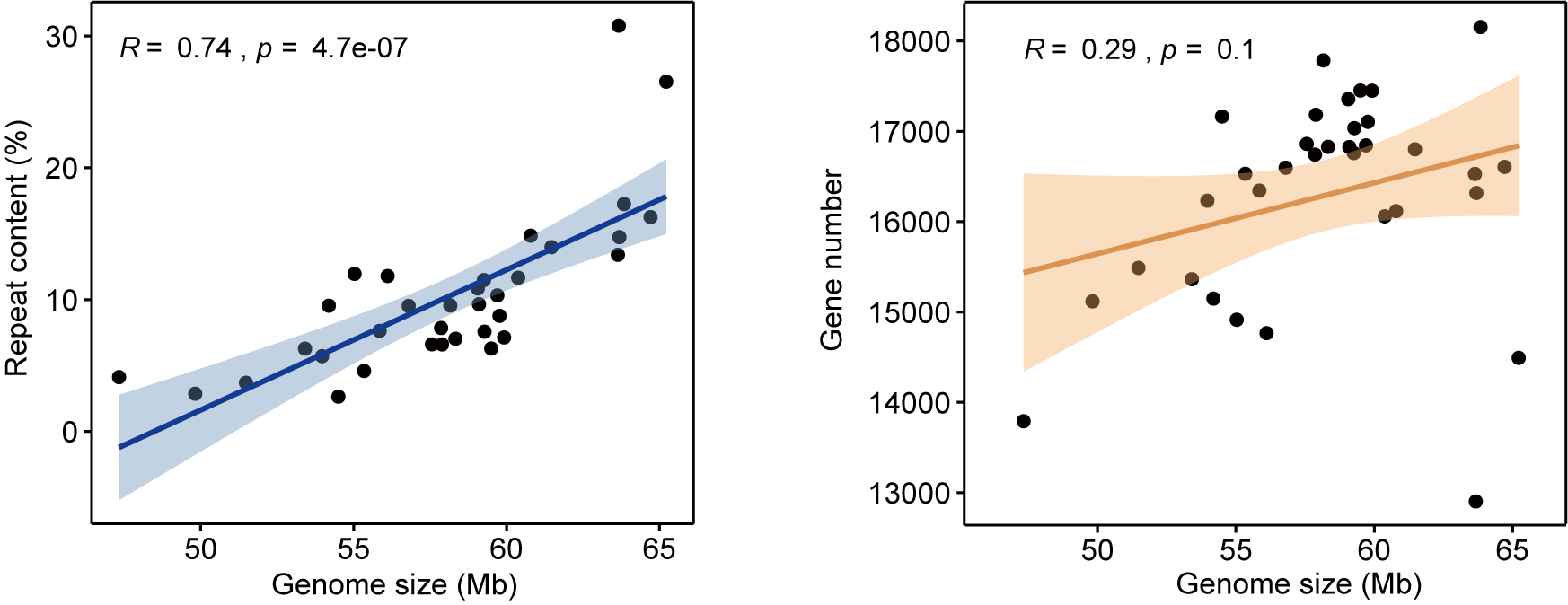
Figure S3. Correlation of genome size with repeat content and gene number across *Diaporthe* genomes.** Scatter plots illustrate the relationships between genome size and (left) total repetitive content and (right) annotated gene number across *Diaporthe* strains. *R*, Pearson correlation coefficient.


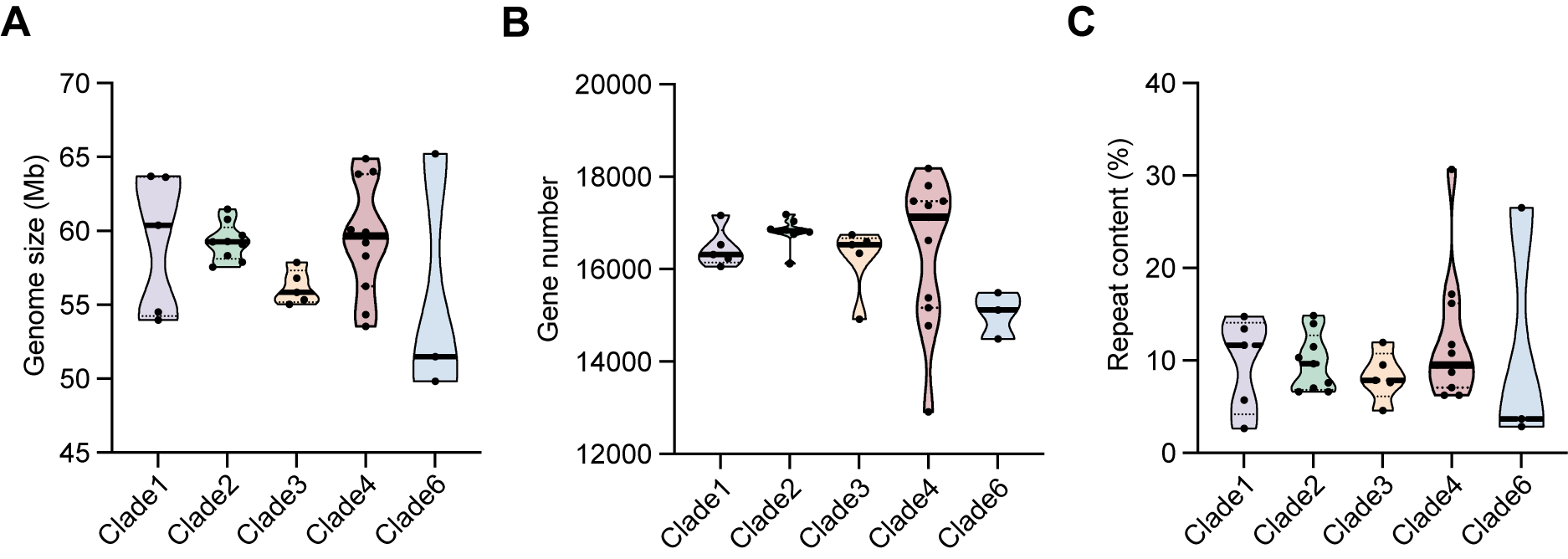


**Figure S4.** **Comparative analysis of genomic features across *Diaporthe* evolutionary clades.** A) Genome size. B) Number of predicted protein-coding genes. C) Proportion of repetitive elements in the genome. The box plots represent median, interquartile range, and data range across clades.


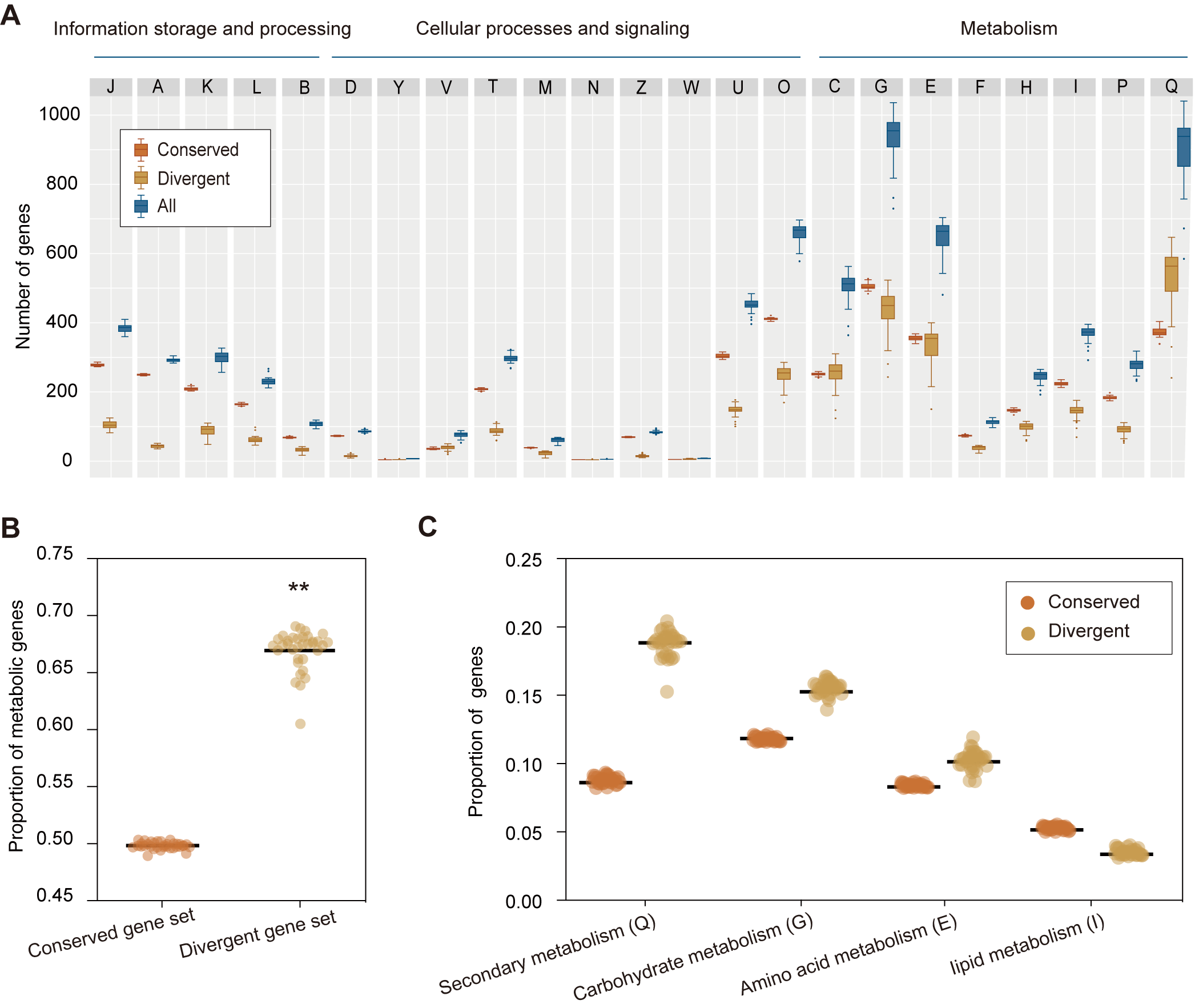


**Figure S5. Functional classification of *Diaporthe* genomes based on KOG annotation.** A) Distribution of predicted proteins assigned to functional categories defined by euKaryotic Orthologous Groups (KOGs). B) Proportion of metabolism-related genes in the conserved gene set versus the divergent gene set, which highlighting functional divergence across gene compartments. Statistical significance is indicated by asterisks (***P* < 0.01, two-sided Wilcoxon tests, n = 34). C) Detailed breakdown of metabolic genes categorized by KOG subcategories.


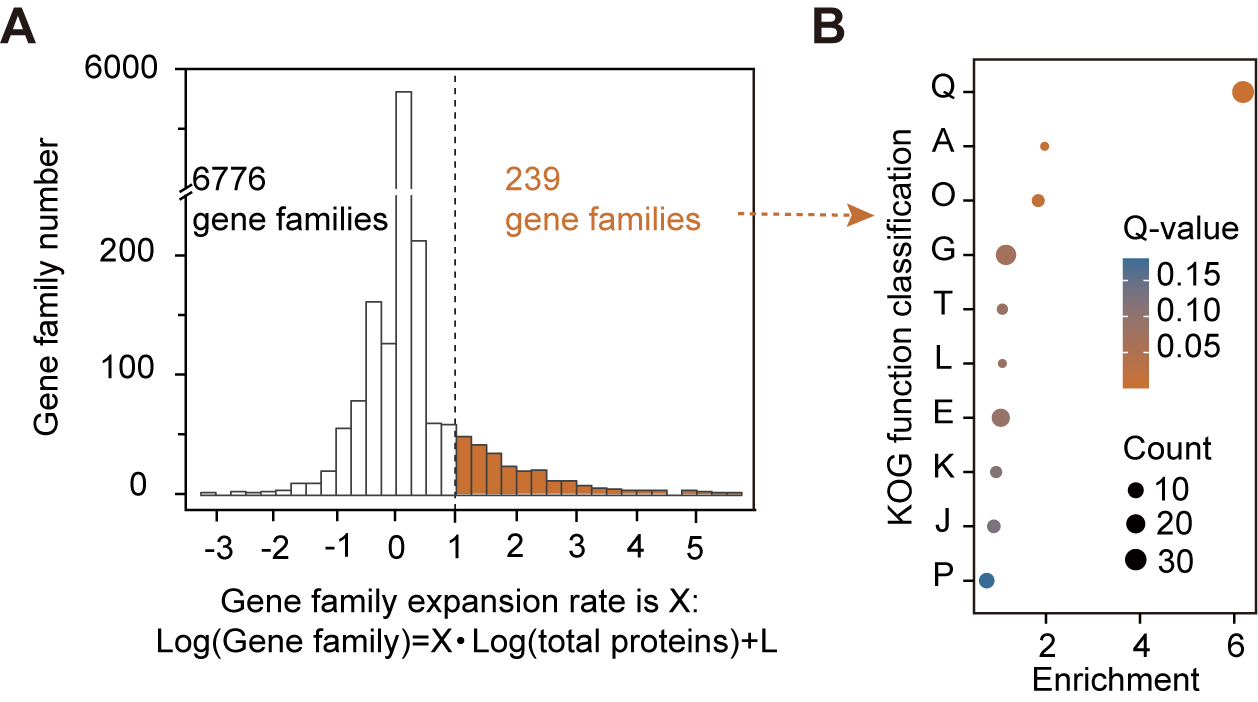


**Figure S6.** **Expansion of gene families in *Diaporthe* genomes.** A) Histogram showing the distribution of gene family expansion rates across 34 *Diaporthe* genomes. The expansion rate was calculated using the formula displayed in the panel. B) Functional enrichment analysis of gene families exhibiting significant copy number expansion.


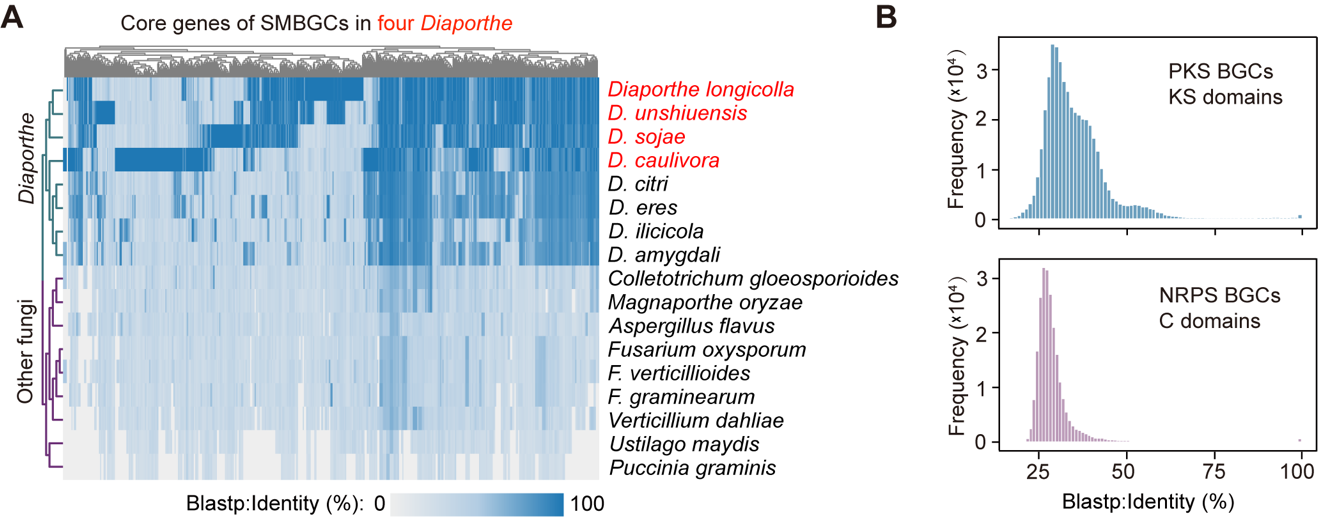


**Figure S7.** **Sequence conservation of SMBGC core genes and biosynthetic domains across fungal genomes.** A) Heatmap showing the sequence identity of four *Diaporthe* SMBGC core genes compared against other fungal genomes. B) Distribution of sequence identity for biosynthetic marker domains across predicted PKS and NRPS clusters. Top: ketoacyl synthase (KS) domains in PKS clusters. Bottom: condensation (C) domains in NRPS clusters.

**
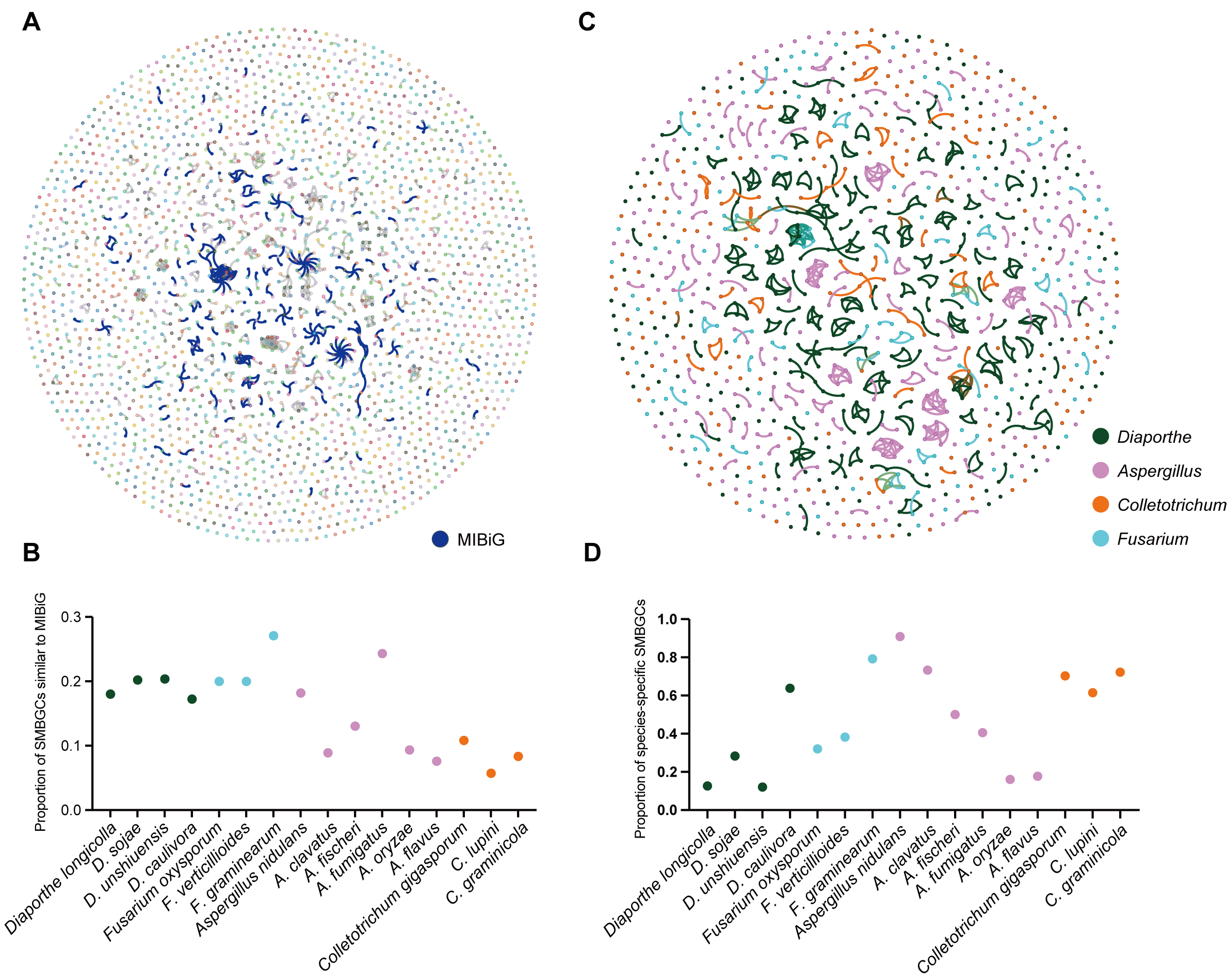
**

**Figure S8. Comparative similarity network analysis of SMBGCs across fungi.** A) Similarity network of SMBGCs from non-*Diaporthe* fungi. Each node represents an individual SMBGC. SMBGCs annotated in the MIBiG database are shown in blue, while SMBGCs from different fungal species are indicated by distinct colors. Edges connect SMBGCs exhibiting sequence similarity. B) Proportion of SMBGCs in representative fungal species that show similarity to known SMBGCs in the network. C) Similarity network of SMBGCs from representative fungal species. D) Proportion of species-specific SMBGCs in representative fungal species identified in the network.


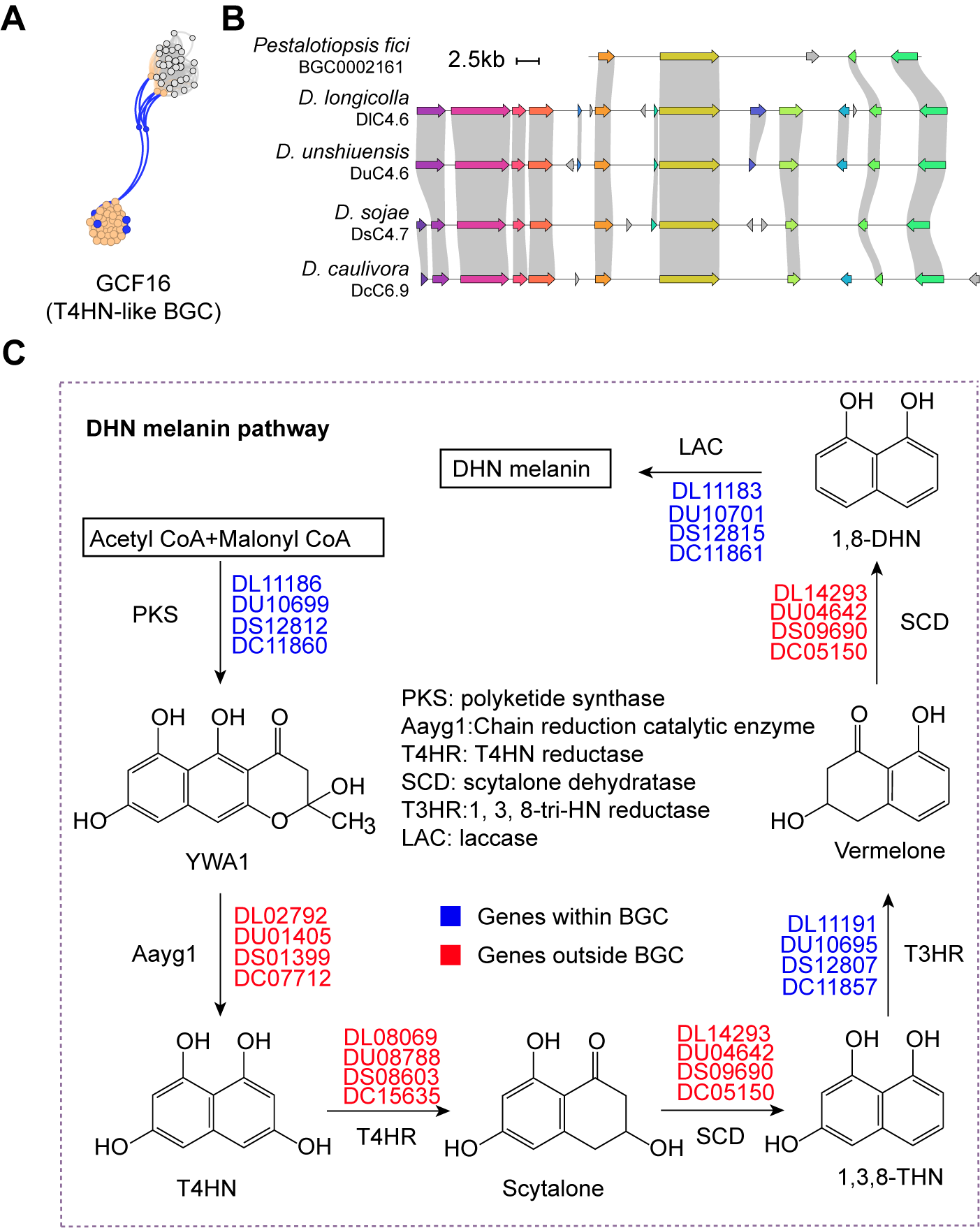


**Figure S9.** **Features of T4HN-like BGCs and melanin biosynthesis pathways*.*** A) Gene cluster family (GCF) analysis identifies a conserved T4HN-like biosynthetic gene cluster (GCF16) shared among *Diaporthe* species and *Pestalotiopsis fici*. B) Collinearity analysis of T4HN-like BGCs in *Diaporthe* and *Pestalotiopsis fici*. C) Reconstruction of the DHN (1,8-dihydroxynaphthalene) melanin biosynthetic pathway in *Diaporthe*, based on homology to known pathway genes. Genes within the T4HN BGC are shown in blue; homologs located outside the BGC are shown in red.


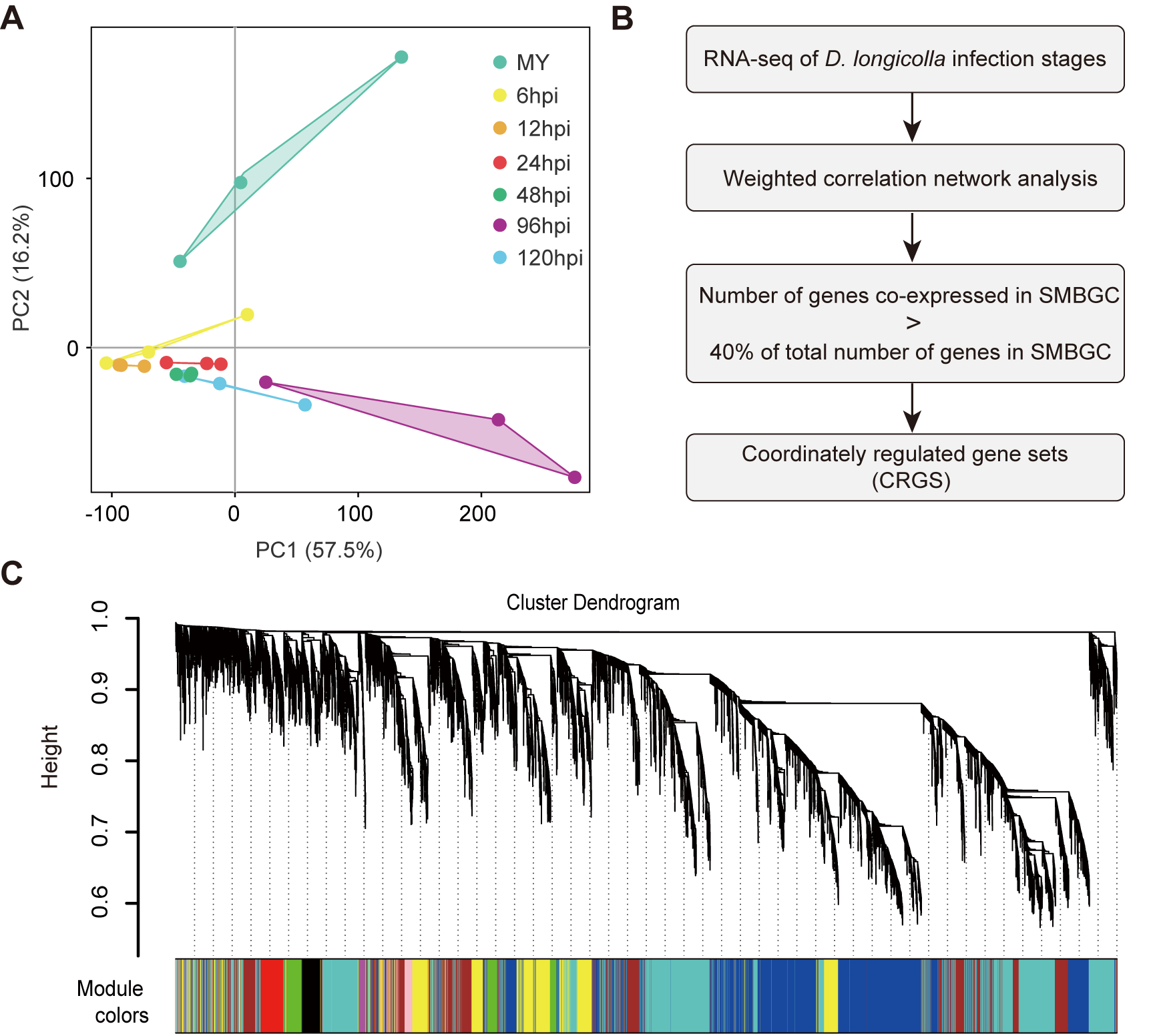


**Figure S10.** **Sample clustering and weighted gene co-expression network analysis (WGCNA) of transcriptomic data.** A) Principal component analysis (PCA) of transcriptome sequencing samples. MY represents the mycelial stage samples, while 6-120 hpi represents samples from different hours of infection. B) Workflow for identifying SMBGCs classified as coordinately regulated gene sets (CRGS). SMBGCs in which ≥40% of genes are co-expressed within a single module were defined as CRGS. C) Gene dendrogram generated by WGCNA. Modules representing clusters of co-expressed genes are shown in distinct colors.


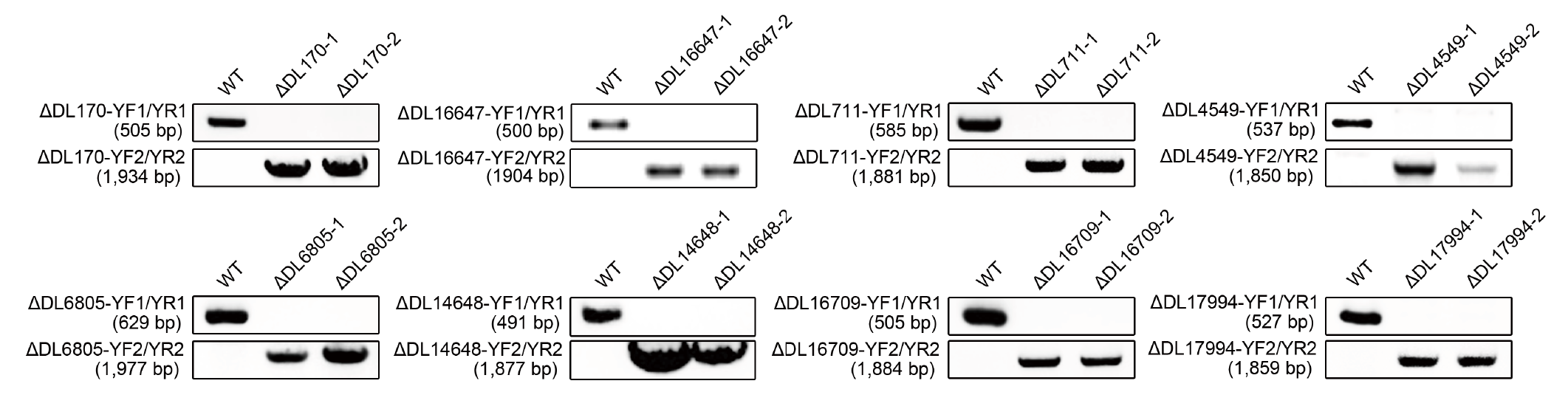


**Figure S11.** **PCR verification of gene deletions in *D. longicolla* mutants.** PCR assays confirm the presence or absence of target bands in wild-type (WT) and corresponding gene deletion mutants (Δ). Each panel displays the expected band size and genotype-specific amplification pattern for two independent transformants.

**
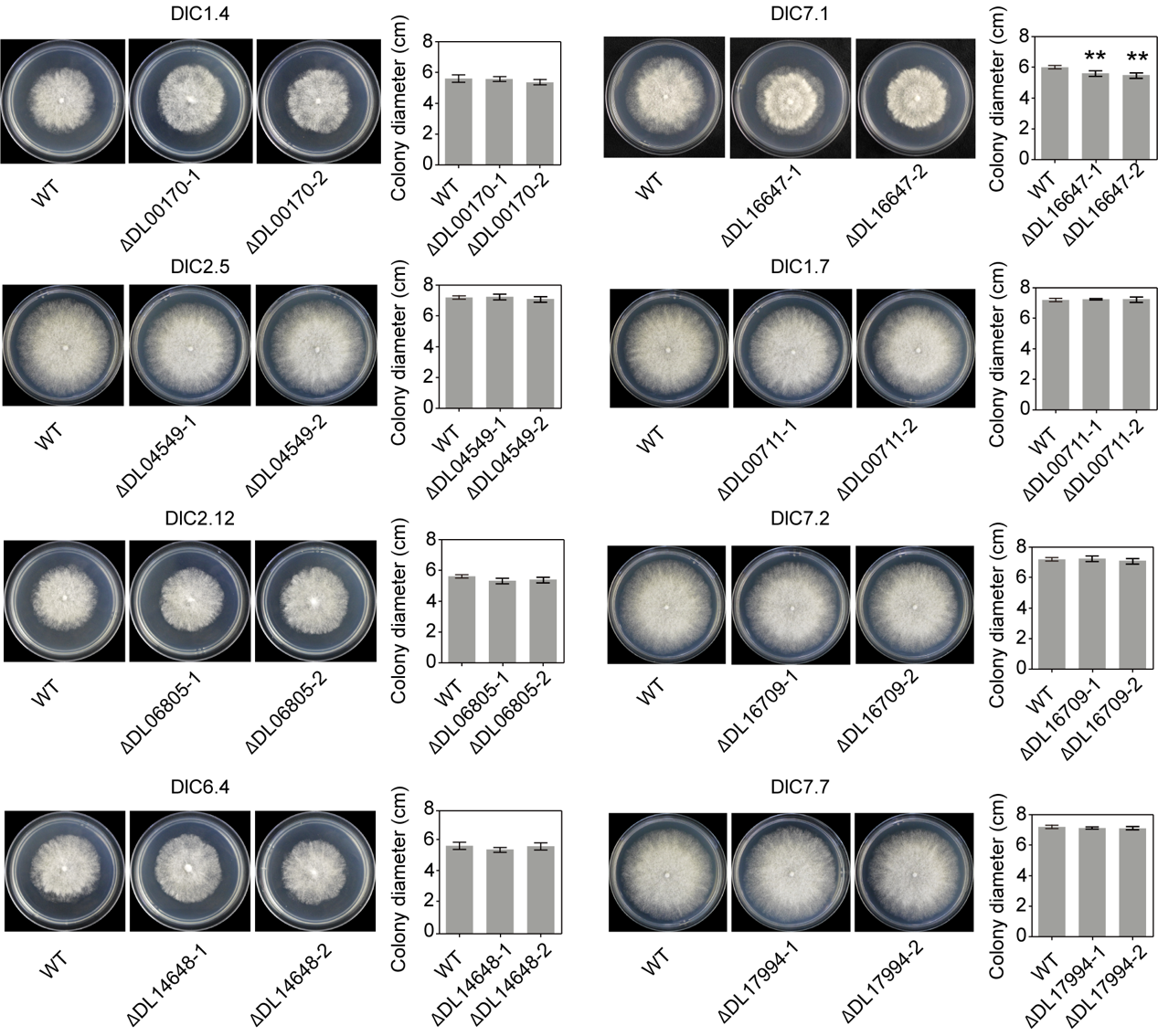
**

**Figure S12.** **Impact of SMBGC core gene deletions on vegetative growth in *D. longicolla*.** Colony morphology and diameter of wild-type (WT) and gene deletion mutants (Δ) grown on CM plates for three days. Representative images and quantitative measurements are shown for each SMBGC core gene mutant. Asterisks indicate significant differences in colony diameter relative to WT (***P* < 0.01, two-sided Wilcoxon tests, n = 3).


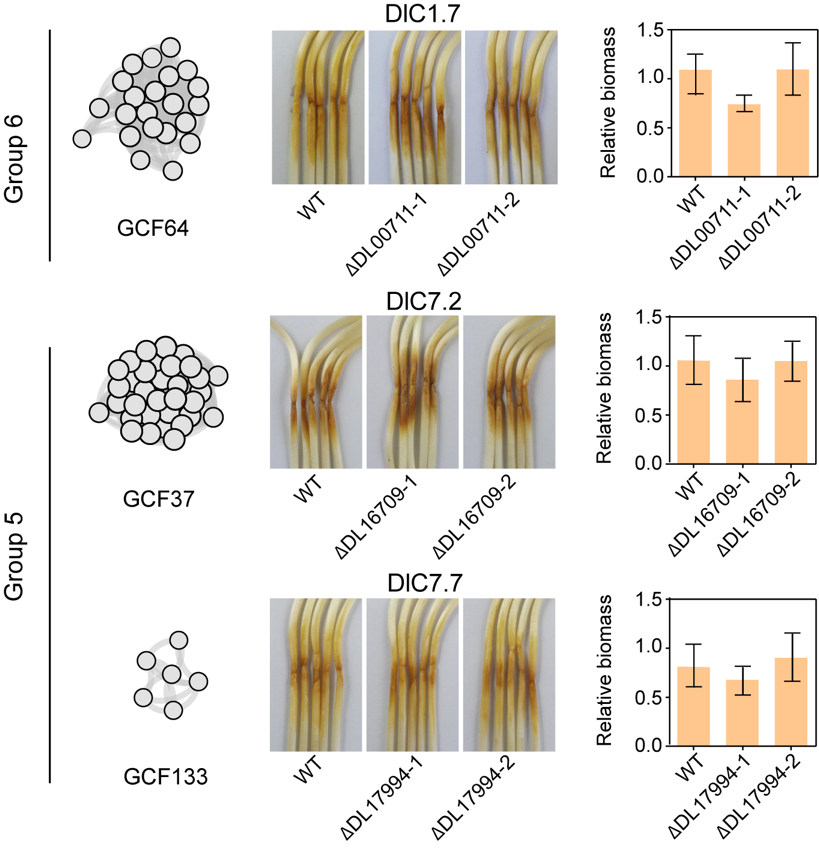


**Figure S13.** **Knockout of three SMBGCs does not affect virulence of *D. longicolla*.** Representative infection phenotypes in soybean and corresponding relative biomass measurements of wild-type (WT) and gene deletion mutants (Δ). No significant differences in pathogenicity were observed between WT and mutants based on fungal biomass quantification (*P* > 0.05, two-sided Wilcoxon tests, n = 3).

**
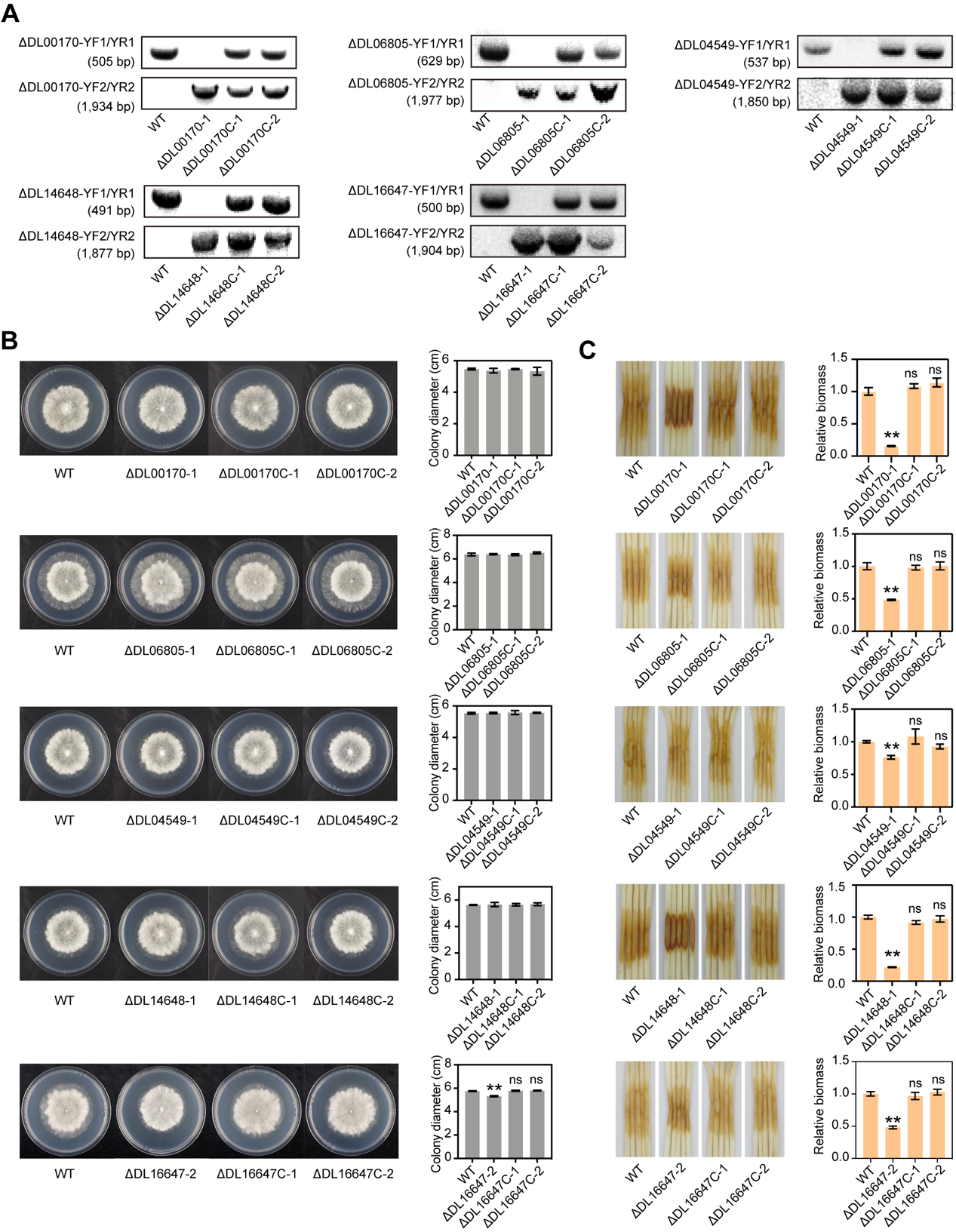
Figure S14. Functional validation of gene deletion mutants through genetic complementation.** A) PCR verification of successful re-introduction into *D. longicolla* deletion mutants. PCR assays confirm the presence of target bands in wild-type (WT) and complementation mutants (Δ-C), and their absence in the corresponding gene deletion mutants (Δ). Each panel displays the expected band size and genotype-specific amplification pattern for two independent complementation transformants. B) Impact of gene complementation on vegetative growth in *D. longicolla*. Colony morphology and diameter of wild-type (WT), deletion mutants (Δ) and complemented strains (Δ-C) grown on CM plates for 3 d are shown. Asterisks indicate significant differences in colony diameter relative to WT (***P* < 0.01, two-sided Wilcoxon tests, N = 3). C) Complementation assays restore full virulence. Pathogenicity tests on soybean demonstrate that while virulence is attenuated in the deletion mutants, all complemented strains exhibit pathogenicity levels comparable to the WT. (***P* < 0.01, two-sided Wilcoxon tests, n = 3).


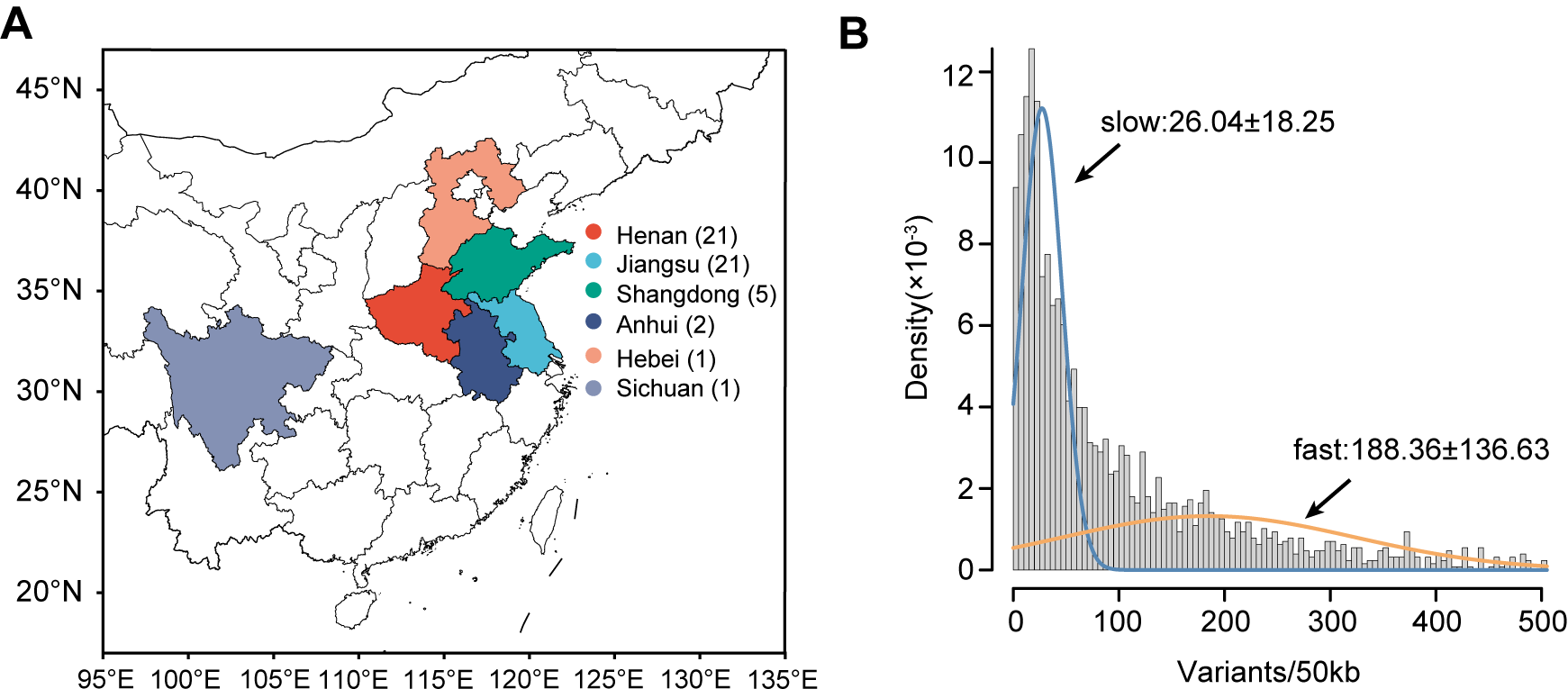


**Figure S15. Geographic distribution and genomic variation of *D. longicolla* isolates.** A) Sample collection site for isolating 51 *D. longicola* strains. The boundaries of China’s administrative divisions were obtained from the National Catalogue Service for Geographic Information (www.webmap.cn; approval number GS (2024) 0650). The base map has not been modified. B) Histogram of variant density distributions for *D. longicolla* YC2-1 based on a bin of 50 kb. The curves illustrate the two Gaussian distributions estimated based on a Gaussian mixture model using the Expectation-Maximization algorithm.


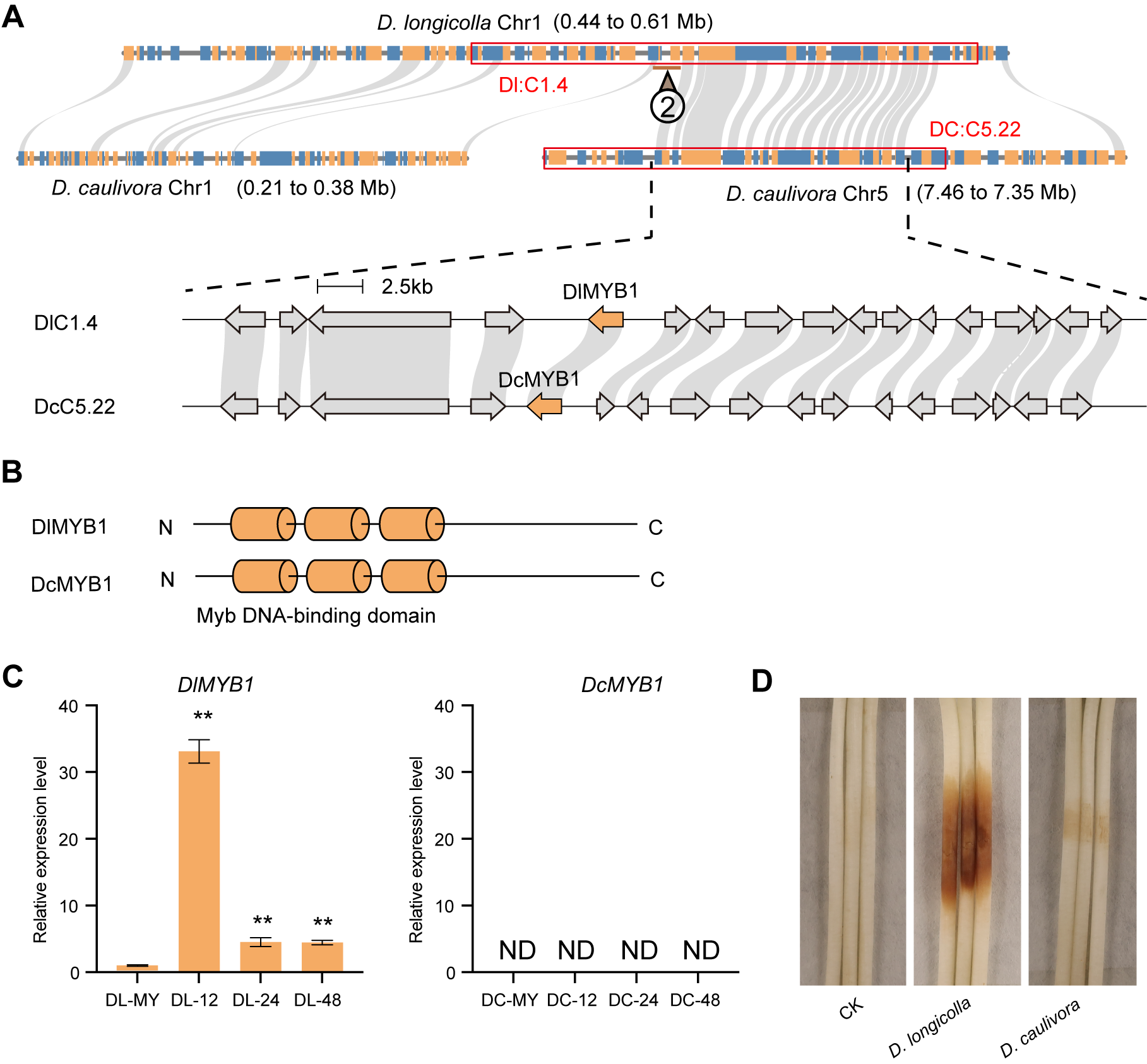


**Figure S16. Expression divergence and structural conservation of MYB1 transcription factors between *D. longicolla* and *D. caulivora.*** A) Synteny analysis of SMBGCs DlC1.4 and DcC5.22 reveals conserved genomic regions between *D. longicolla* Chr1 and *D. caulivora* Chr5, containing homologous MYB1 transcription factors. B) Protein domain organization of DlMYB1 and DcMYB1. Both contain conserved Myb DNA-binding domains. C) Relative expression of *DlMYB1* and *DcMYB1* at different time points post inoculation in soybean, normalized to the mycelial stage (MY). ND (Not Detected) indicates samples with CT values >35, reflecting transcript levels below the detection limit. Statistical significance indicated by asterisks (***P* < 0.01, two-sided Wilcoxon tests, n = 3). D) Infection phenotypes of soybean stems inoculated with *D. longicolla* YC2-1 and *D. caulivora* SQ1-13*,* compared to the mock control (CK).


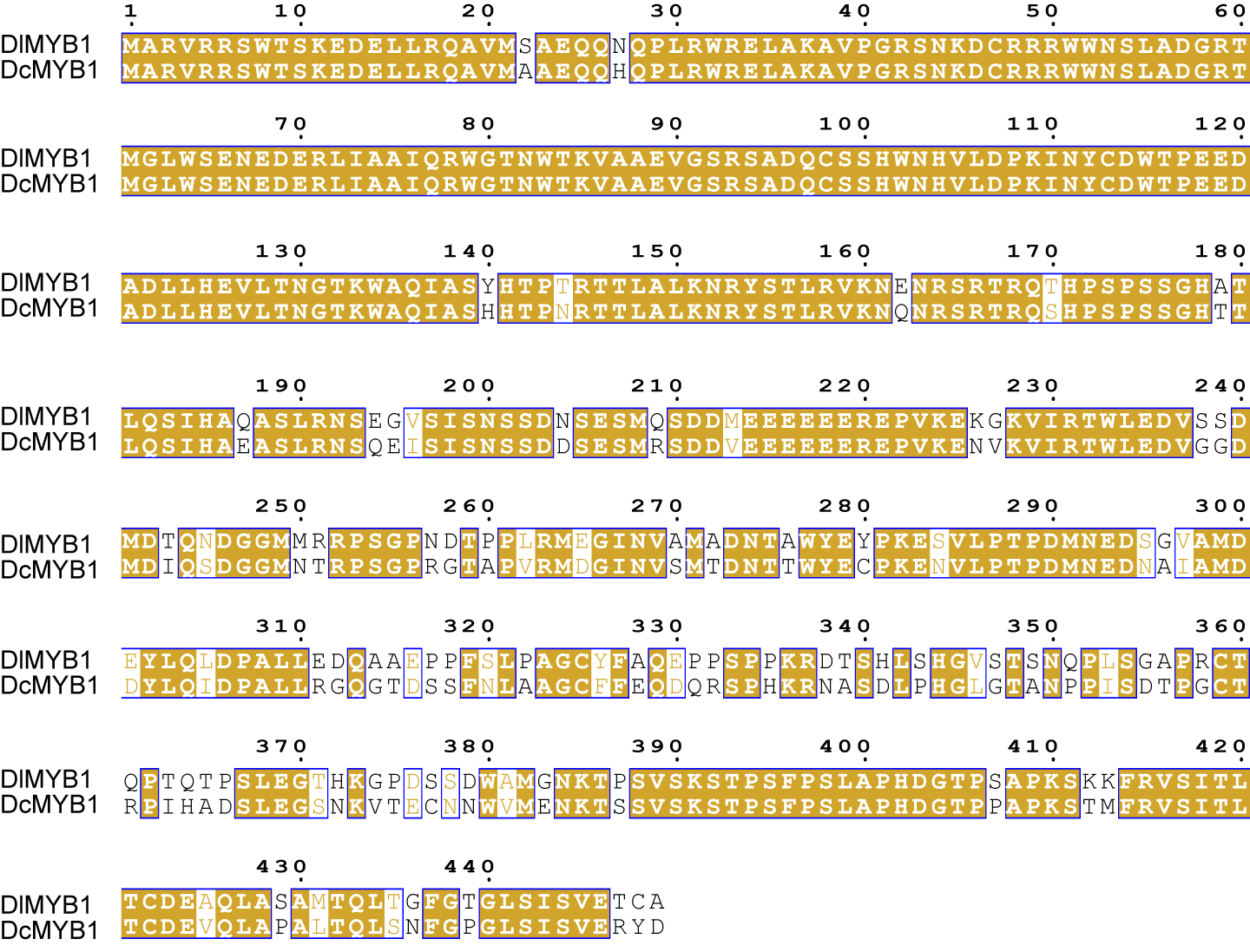


**Figure S17.** **Protein sequence alignment of MYB1 from *D. longicolla* and *D. caulivora*.**


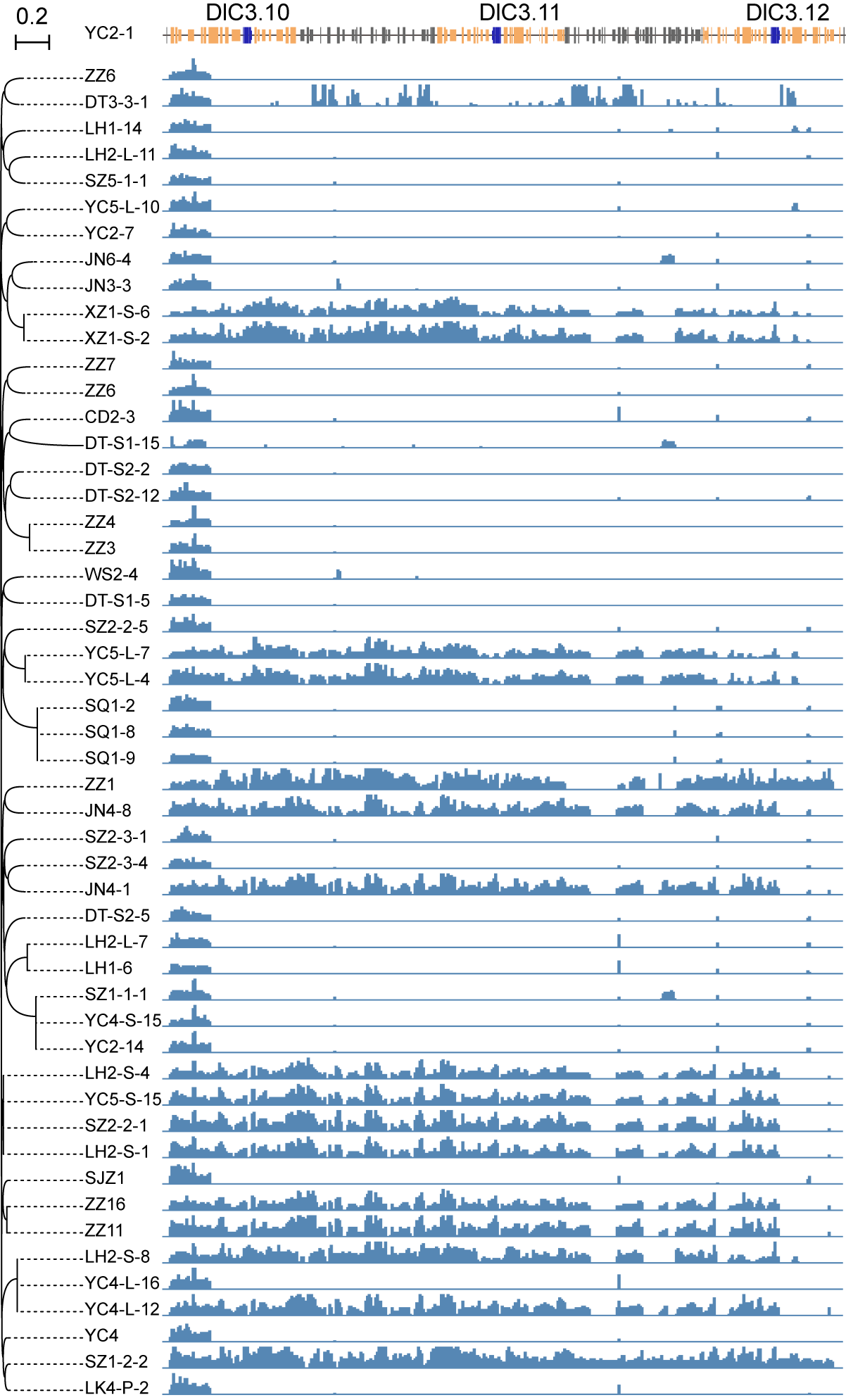


**Figure S18. Strain-level variation in the presence of SMBGCs across 51 *D. longicolla* isolates.** Phylogenetic tree of 51 *D. longicolla* strains constructed based on genome-wide SNPs (left), alongside presence–absence profiling of three adjacent SMBGCs (DlC3.10, DlC3.11, DlC3.12; top).


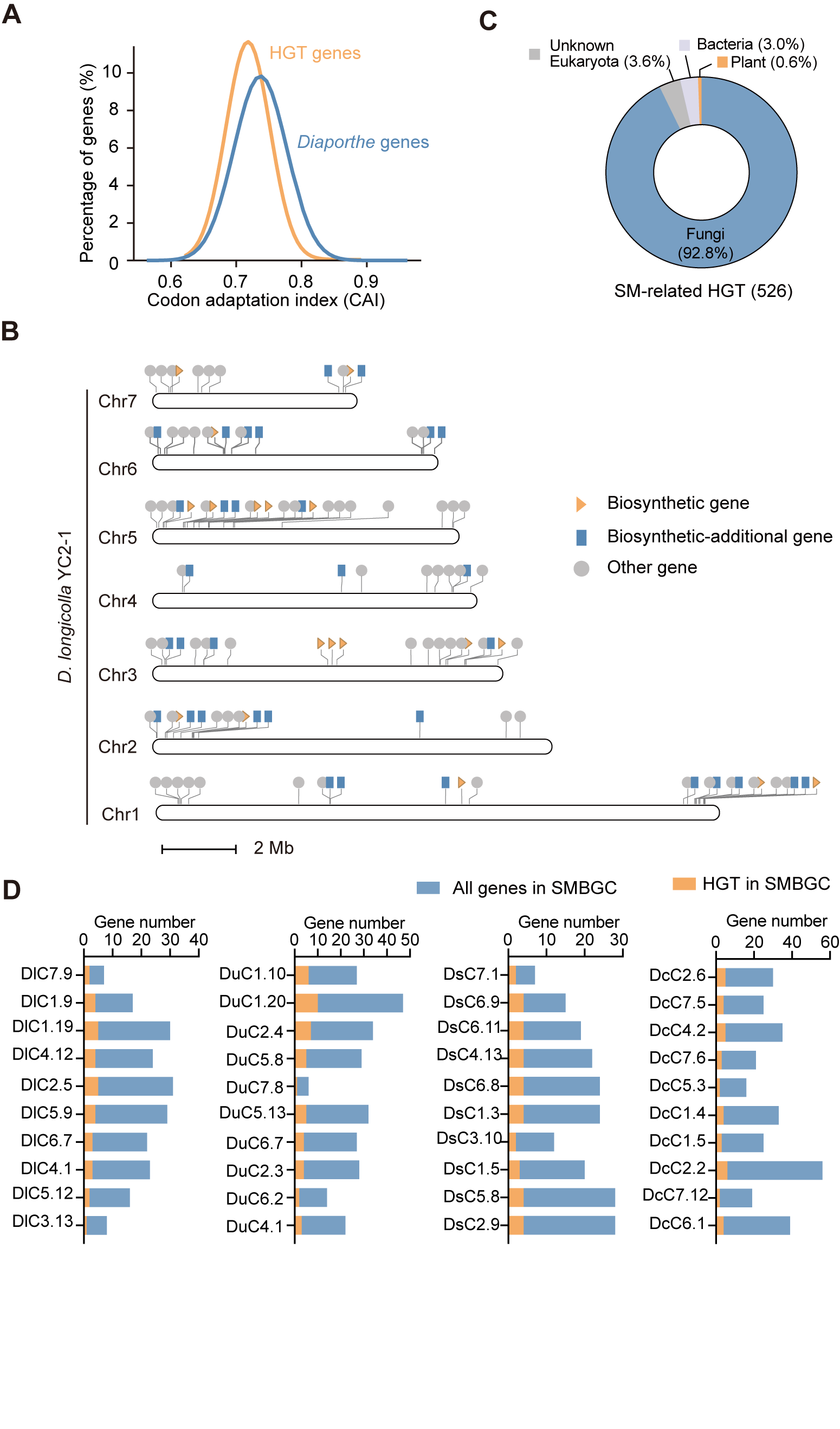


**Figure S19. Prediction and annotation of horizontally transferred genes in *Diaporthe*.** A) The Codon Adaptation Index (CAI) distribution of HGT genes compared to *Diaporthe* genes. The CAI derived from the RSCU estimations is computed using the EMBOSS tool “cai”. B) Chromosome distribution of SMBGCs related HGT in *D. longicolla* prediction. C) Predicted donor distribution of HGT associated with SMBGCs. D) The ten SMBGCs exhibiting the highest proportions of putative HGT-derived genes are shown
